# Synthetic wheat as a new source of flour quality under drought conditions: Associations with solvent retention capacity

**DOI:** 10.1371/journal.pone.0316945

**Published:** 2025-02-06

**Authors:** Niloofar Mokhtari, Mohammad Mahdi Majidi, Aghafakhr Mirlohi

**Affiliations:** Department of Agronomy and Plant Breeding, College of Agriculture, Isfahan University of Technology, Isfahan, Iran; Institute of Genetics and Developmental Biology Chinese Academy of Sciences, CHINA

## Abstract

Synthetic hexaploid lines are proposed as high-potential germplasm for improving bread wheat by introducing new genes (biotic and abiotic stresses) lost during common wheat evolution. A panel of 99 synthetic and common wheat was studied for quality and grain-related traits and drought tolerance under two different moisture conditions (water stress and normal) during two growing seasons. Results indicated different variations for most traits suggesting that the synthetic hexaploid wheat-derived lines (SHW-DL) panel contains valuable genes for drought tolerance improvement of wheat. Drought stress reduced morphological traits and production but increased protein (Pro), rapid mix test (RMT), and solvent retention capacity (SRC) traits. Synthetic wheat lines were superior with higher grain yield, glutenin, damaged starch, available pentosane, overall water holding capacity, and gluten strength (glutenin and gliadin strength) compared to common wheat making them more suitable for bread-baking. The results showed that solvent retention capacity had a strong capacity to differentiate the quality of wheat genotypes. Correlation analysis indicated that genetic improvement for high-yielding varieties can be achieved by producing more damaged starch, higher water absorption, hardness, and lower gluten strength, and zeleny (ZEL). Selection of superior genotypes using univariate and multivariate methods will be discussed.

## Introduction

Common wheat (*Triticum aestivum* L.) is one of the most widely grown cereal crops and the primary source of food [1]. Bread wheat is the consequence of combining *T*. *urartu* (donor of A genome), *Aegilops speltoides* (donor of B genome), and *Ae*. *tauschii* (donor of D genome) [2]. Due to domestication and modern breeding, a genetic bottleneck has occurred, resulting in a limited genetic pool of wheat germplasm [3]. To compensate for the narrow genetic diversity of bread wheat, SHW was produced from the combination of novel and elite genes from the tetraploid wheat (*Triticum turgidum* L. (AABB)) and wild ancestors and landraces (*Ae*. *tauschii* (DD), then crossing SHW lines with different elite ones by varying levels of genetic introgression from the ancestors [2]. Recently, SHW-derived lines (SHW-DL) have been proposed as a suitable germplasm for environments affected by drought and high temperatures caused by living and non-living stress factors, because of an increase in level of photosynthesis and notable growth in grain production [3,4]. Using root morphological traits, Becker et al. (2015) found that extracting water from deeper soil layers under drying conditions led to an increase in productivity in SHW-DL compared to winter wheat [4]. Additionally, it is indicated that SHW-DL has higher antioxidant activities and better agronomic performance [5], demonstrating the genetic capacity of synthetic wheat to enhance yield and yield consistency in dry, and high-temperature environments.

Drought is an abiotic constraint that affects crop productivity, which can cause losses in performance and growth at different stages of wheat, with significant impact during anthesis [6]. In this respect, determining drought-tolerant varieties of high quality among the wheat population is a prerequisite for the future. Previous research found that larger grains and higher grain-filling rates have been produced by relatively higher-yielding cultivars in low-yielding environments [7]. It was also found that the heat-susceptibility index under high-temperature conditions is negatively correlated with grain yield, chlorophyll content, and kernel weight of synthetic wheat lines [8]. Applying appropriate drought and susceptibility indices may also improve the efficiency of selecting superior genotypes under stress conditions. Among the indices applied, the combination of best-identified tolerance and susceptibility indices seem to be the most effective indicators for screening superior lines [9]. The bread-baking ability differs amongst different genotypes. Hence, determining the flour components, quality, and functionality is vital along with grain yield, and its drought tolerance [10].

Flour quality may be influenced by various factors, including plant genetics, agronomic practices, and processing techniques [10]. Numerous methods are used to evaluate flour quality. The SRC is a new and unique method that was developed by Louise Slade [10]. SRC was used to measure flour functionality based on the expansion characteristics of polymerized flour constituent structures with the least amount of flour (below 1 g). Using the SRC profile, the characteristics of baking and processing can be predicted. The SRC test evaluates important aspects of wheat quality including general water retention (solvents-water) (WSRC), glutenin network formation, and gluten elasticity (using 5% w/w lactic acid (LA) in water) (LASRC), damaged starch (using 5% w/w sodium carbonate (Na_2_CO_3_) in water) (SCSRC), and pentosan and gliadin content (using 50% w/w Sucrose in water) (SuSRC) [10,11]. To assess the specific impact of gluten subunits, four additional SRC solvents have been suggested. Furthermore, the functionality of particular gluten components has been evaluated using supplemental liquids (0.006%-sodium metabisulfite (NaMBS), 0.75%-sodium dodecyl sulfate (SDS), 0.75%-SDS + 0.006%-NaMBS, 55%-ethanol, and CaCl_2_). Different characters have been measured using these solvents including the general strength properties of gluten with MBSSRC, glutenin macropolymer (GMP) with SDS liquid, GMP without S–S network by SDS+MBS, gliadins [12], and beta-glucans with EtSRC [13]. The general function of gluten in the presence of other influencing networks has been evaluated using the SRC gluten-performance-index (GPI) value [14]. Recently, SRC has been applied by several researchers to predict the flour functionality of different types of wheat for various uses due to its rapid, low-cost, and reliable tests [15–17]. Some flour quality parameters such as protein, moisture, ZEL, hardness (HAR), RMT, and water absorption can be determined by Near-Infrared Reflectance (NIR). NIR is an instrumental method used to analyze flour rapidly instead of employing chemical techniques. The NIR technique has already been applied in various aspects, including differentiating wheat genotypes at different times, identifying the geographical origins, and classifying wheat varieties [16,18].

The development of new wheat varieties suited to different stress environments is inevitable because of minimal genetic variation within wheat germplasm and the prospect of increased drought events which can be worsen by the climate change. Consequently, many researchers suggest SHW-DL to address these issues because it represents a broader genetic foundation than diploid and tetraploid wheat relatives [8]. Several SHW-DL varieties have been developed at CIMMYT that are available for testing in different regions under various conditions to explore their genetic potential in replacing common wheat cultivars [4,9]. With the advent of the SRC technique, it is now possible to screen large panels of SHW-DL wheat for quality attributes grown in different environments and to select higher-yielding genotypes with improved bread-baking flour qualities. Coupled with the Near Infrared Reflectance (NIR) technique, SRC provides an excellent opportunity for screening a large number of samples with high reliability, low cost, rapid, and small sample size. A study has yet to determine the flour quality related to SHW-DL under water stress and to compare it with common wheat. Hence, the aims of the present project were to 1) assess a large panel of SHW-DL grown under water stress conditions based on the grain yield, the components of yield, and the quality flour and 2) recognize the potential lines for drought tolerance with a suitable quality of flour through univariate and multivariate methods.

## Material and methods

### Plant material and experimental site

Before initiating the fieldwork, approval for the conduct of the survey was obtained from ethics committee of Isfahan University of Technology as well as University of Guelph by Dr Ali Navabi. Approval of the survey by these four ethics Committees required submitting all survey material to their members for evaluation.

Synthetic hexaploid wheat lines were produced in the gene bank of the International Maize and Wheat Improvement Center (CIMMYT). Each line was developed by hybridizing 13 different tetraploid wheat genotypes with 19 distinct *Ae*. *tauschii* accessions producing 23 main SHWs (S1 Table). The development of synthetic wheat lines has involved making one to five crosses with elite hexaploid wheat, from 2^nd^-degree to 5th-degree synthetic (primary synthetic crossed with one and four common hexaploid lines, respectively). The current research assessed 99 wheat varieties, comprising 91 SHW-DL selected from a broad germplasm pool and eight bread wheat cultivars (including AAC Scotia, Carbery, Norwell, and Sable from Canada and Pishtaz, Qhods, Kavir, and Roshan from Iran) as control (S2 Table), across two distinct moisture conditions of water stress and normal.

The experiment was carried out over two winter cropping periods (2018–2020) at Isfahan University of Technology’s Research Farm (32° 30′ N, 51° 20′ E). The soil had a silty clay loam texture with a pH 8.3. The basic 11 × 11 lattice square design was applied with two repetitions and included additional genotypes as fillers.

### Growth treatments

Planting rows were 2m long with a spacing of 20cm, leading to a seeding density of 300 seeds per square meter according to previous research (9). In both years, plants were sown under two different conditions (normal and stressed). Water depletion of 45% and 90% at field capacity (FC) was applied to provide two moisture environments during the flowering stage.

### Measurement of traits

Protein (%), moisture (%), zeleny (mL), water absorbance (mL), hardness (%), and RMT (mL /100 g flour) were measured using near-infrared reflectance. The Solvent Retention Capacity (SRC) test was conducted following Guzman et al. (2015) with some modifications to method 56–11.02-AACC, 2000 [19]. The four standard solvents (water (WSRC), lactic acid (LASRC) (5%), sodium carbonate (SCSRC) (5%), and sucrose (SuSRC) (50%)) and the five supplementary solvents (sodium dodecyl sulfate (SDSSRC) (0.75%), sodium metabisulfite (MBSSRC) (0.006%), 0.75% SDS-SRC+0.006% NaMBS (SDS+MBSSRC), ethanol (EtSRC) (55%) [12], and calcium chloride (CaCl_2_SRC) (5%) [13] were utilized. In the scaled version of the SRC method, 0.3 g of flour samples were placed into previously weighed tubes and mixed with each solvent (1.5 mL) using a vortex. Immediately, the tubes were shaken for 5 minutes (25°C) and then centrifuged for 2 minutes at 4000g. After centrifugation, the supernatant was omitted and the tubes were drained for 10 minutes on tissue paper. Finally, the weight of the tubes was measured, and the two indices, including (SRC and GPI) were calculated according to the Formulas 1, 2 [10]:

SRC(%)=[tubeandgelweight‐emptytubeweight/flourweight][(86/100‐flourmoisture)‐1]*100 (1)

GPI=[LASRC/(SuSRC+SCSRC)]*100 (2)

Thousand grain weight (TGW g), hectoliter (He Kg/he), grain yield (GY g/m^2^) and drought tolerance indices (STI, YSI, and CSI) were measured after grain harvest according to previous research [9].

### Statistical and genetic analyses

The normal distribution of the data was examined using SAS 9.440 software. To select the appropriate design for analysis, the relative efficiency of the experimental design was measured indicating that Lattice design was less than the randomized complete block design (RCBD). Therefore, the RCBD procedure was chosen to analyze the data. An analysis of variance (ANOVA) was conducted to partition the variation into components in SAS 9.440 software [20] with the least significant difference (LSD) value at the 5% probability level. The correlation among the quality traits and yield was calculated using PROC CORR for two environments (normal and water stress), separately. Based on a correlation matrix, principal component analyses (PCA) were performed separately for both environments. An SRC quality index (SQI) (Eqs 3 and 4) was created using the first principal component analysis of SRC-related traits as follows. Additionally, the NIR quality index (NQI) (Eq 5) was created using the first principal component analysis of NIR-related traits as follows.

$$For\;normal\;conditions:\;SQI=(0.786*WSRC)+(0.892*SCSRC)+(0.823*SuSRC)+(0.861*LASRC)+(0.890*MBSSRC)+(0.893*Cacl_{2}SRC)+(0.837*SDSSRC)+(0.841*SDS+MBSSRC)+(0.859*EtSRC)$$ (3)

$$For\;water\;stress\;conditions:\;SQI=(0.787*WSRC)+(0.923*SCSRC)+(0.936*SuSRC)+(0.918*LASRC)+(0.917*MBSSRC)+(0.932*Cacl_{2}SRC)+(0.876*SDSSRC)+(0.936*SDS+MBSSRC)+(0.744*EtSRC)$$ (4)

Fornormal&waterstressconditions:NQI=(0.876*Pro)+(0.734*ZEL)+(0.926*WABS)+(0.906*HAR)+(0.807*RMT)+(0.903*MOI) (5)

Genetic coefficient of variation (GCV) and heritability was calculated based on the previous research (9).

## Results

### Analysis of variation and variation in wheat quality parameters

Variance analysis revealed significant impacts of moisture conditions on the examined traits (*P* < 0.05) (except for moisture, zeleny, water absorbance, and hardness) (S3 Table). The existence of significance for the genotypic effect indicated variation among genotypes (*P* < 0.01) (S3 Table). Significant variations existed among each planting year for moisture, zeleny, water absorbance, hardness, RMT, GY, and LASRC (S3 Table). The range of GCV was from 1.18 (GPI) under normal conditions to 24.63 (zeleny) under stress conditions. The variations of some traits, including protein, moisture, water absorbance, hardness, and grain yield under normal conditions were higher than water stress conditions, whereas the remaining traits were higher under water stress conditions. The range of broad sense heritability was from 15.84 (GPI) to 93.08 (protein) for normal conditions and from 17.83 (RWC) to 94.85 (EtSRC) for the water stress conditions. The traits with higher heritability included RMT, TGW, GY, He, WSRC, SCSRC, SuSRC, LASRC, MBSSRC, Cacl_2_SRC, SDSSRC, SDS+MBSSRC, EtSRC, and GPI under water stress environments (Table 1). The heritability of grain yield was moderately low (32.24).

**Table 1:** Descriptive information of wheat quality and agronomy traits evaluated under normal and water-stressed conditions during two years (R: Range, GCV: Genotypic coefficient of variation, h^2^: Broad sense heritability).

Traits	R (Min-Max)		GCV (%)		h^2^		
	Normal	Stress	Normal	Stress	Normal	Stress	Total
**Protein(%)**	12.80–20.80	14.30–22.20	18.13	13.68	93.08	90.63	95.80
**Moisture(%)**	6.70–10.00	6.50–9.80	4.99	4.16	41.28	36.62	53.06
**Zeleny(mL)**	24.00–107.00	28.00–106.00	2.66	24.63	89.46	76.93	91.16
**Water Absorbance(mL)**	60.00–71.80	60.00–71.90	3.95	2.62	85.25	64.20	86.67
**Hardness(%)**	47.00–81.00	49.00–78.00	13.15	11.34	88.03	79.01	91.21
**RMT(ml /100 gr flour)**	378.00–1008.00	587.00–1038.00	5.10	19.67	92.78	94.50	96.57
**RWC(%)**	60.78–95.00	40.00–90.00	4.56	7.57	23	17.83	20.69
**TGW(g)**	18.40–67.80	16.21–60.38	11.71	17.47	34.07	37.80	40.73
**GY(g/m** ^**2**^ **)**	232.06–1664.00	202.30–1680.00	13.87	12.95	19.39	21.28	32.24
**He(kg/he)**	54.49–97.50	50.21–92.00	3.95	6.65	29.17	35.97	39.47
**WSRC**	129.29–213.70	178.70–317.92	3.38	12.04	33.60	64.69	65.43
**SCSRC**	192.33–244.71	193.43–268.53	4.19	10.99	54.71	87.57	87.12
**SuSRC**	173.57–253.06	212.29–277.62	5.91	9.78	73.47	85.43	85.94
**LASRC**	182.95–222.01	188.76–253.47	3.27	10.00	51.90	90.78	88.27
**MBSSRC**	174.99–217.35	177.87–285.54	3.16	13.70	38.52	88.76	87.32
**Cacl**_**2**_ **SRC**	194.55–240.93	202.08–275.02	5.32	8.64	71.23	82.79	86.84
**SDSSRC**	167.13–210.47	175.39–241.11	7.14	10.17	75.06	83.13	84.39
**SDS+MBSSRC**	173.46–225.98	176.86–254.68	10.86	14.73	91.38	91.57	94.60
**EtSRC**	160.67–220.83	172.39–254.55	10.31	14.98	87.30	94.85	95.61
**GPI (%)**	41.04–50.07	42.09–51.07	1.18	1.42	15.84	70.77	68.94

RMT rapid mix test (ml/100 gr flour), RWC (%) relative water content, TGW (g) thousand-grain weight, GY (g/m^2^) grain yield, He hectoliter (kg/he), WSRC Water Solvent retention capacity, SCSRC Sodium carbonate Solvent retention capacity, SuSRC Sucrose Solvent retention capacity, LASRC Lactic acid Solvent retention capacity, MBSSRC Sodium Metabisulfite Solvent retention capacity, Cacl_2_SRC Chloride calcium Solvent retention capacity, SDSSRC Sodium dodecyl sulfate Solvent retention capacity, SDS+MBSSRC Sodium Metabisulfite Sodium dodecyl sulfate + Solvent retention capacity, EtSRC Ethanol Solvent retention capacity, GPI Gluten Performance Index.

### Effects of water stress

In 2018, water deficit significantly increased protein, WSRC, SuSRC, LASRC, MBSSRC, SDSSRC, EtSRC, while GPI significantly decreased RWC, TGW, He, and GY (Table 2). In 2019, protein, LASRC, SDSSRC, and EtSRC increased and RWC, GY, and He decreased significantly due to water stress. In both years, the water deficit led to increased protein, LASRC, SDSSRC, and EtSRC. Grain yield showed a wide range of variations (684 g m^-2^–992 g m^-2^ under water stress and normal conditions, respectively). During 2018, WSRC, SuSRC, MBSSRC, and GPI significantly increased under water stress conditions, while they were not affected in 2019 (Table 2).

**Table 2:** Mean of quality traits of SHW-DL evaluated under two irrigation levels during two years.

Traits	2018		2019		Mean of two years	
	Normal	Stress	Normal	Stress	Normal	Stress
**Protein(%)**	16.30^b^	17.95^a^	16.95 ^b^	17.66^a^	16.63 ^b^	17.80^a^
**Moisture(%)**	8.92 ^a^	8.79^a^	7.94 ^a^	7.84^a^	8.43 ^a^	8.32^a^
**Zeleny(mL)**	69.75^a^	71.89^a^	51.12 ^a^	49.37 ^a^	60.43^a^	60.63^a^
**Water Absorbance(mL)**	68.70^a^	68.54^a^	65.42 ^a^	65.51 ^a^	67.06^a^	67.03^a^
**Hardness(%)**	70.01^a^	68.32^a^	60.39^a^	60.07 ^a^	65.20 ^a^	64.19^a^
**RMT(ml /100 gr flour)**	751.70^a^	820.79^a^	807.26^a^	848.87^a^	779.48^b^	834.83^a^
**RWC(%)**	80.53^a^	56.59^b^	78.30^a^	66.85^b^	79.43^a^	61.80^b^
**TGW(g)**	42.39^a^	27.60^b^	38.87^a^	30.32^b^	40.66^a^	29.01^b^
**GY(g/m** ^**2**^ **)**	755.39^a^	484.86^b^	1229.91^a^	878.59^b^	992.07^a^	683.91^b^
**He(kg/he)**	82.98^a^	75.36^b^	84.41^a^	76.93^b^	82.98^a^	75.36^b^
**WSRC**	193.83^b^	208.51^a^	187.42^a^	216.77^a^	190.62^b^	212.64^a^
**SCSRC**	217.18 ^a^	229.10 ^a^	220.81^a^	231.43 ^a^	219.00 ^b^	230.27^a^
**SuSRC**	222.49 ^b^	237.03^a^	229.29^a^	239.43^a^	225.89^b^	238.24^a^
**LASRC**	199.13 ^b^	213.88 ^a^	206.66^b^	216.08^a^	202.90^b^	214.98^a^
**MBSSRC**	194.03^b^	204.82^a^	197.82^a^	206.21^a^	195.92^b^	205.51^a^
**Cacl**_**2**_ **SRC**	221.62^a^	235.62^a^	223.52^a^	230.82^a^	222.57^b^	233.22^a^
**SDSSRC**	186.68^b^	199.89^a^	190.29^b^	203.84^a^	188.48^b^	201.87^a^
**SDS+MBSSRC**	189.53^a^	203.88^a^	192.85^a^	205.88^a^	191.19^b^	204.88^a^
**EtSRC**	189.14^b^	207.39^a^	192.93^b^	209.87^a^	191.04^b^	208.63^a^
**GPI**	45.25^b^	45.70^a^	45.82^a^	45.70^a^	45.63^a^	45.93^a^

RMT rapid mix test (ml/100 gr flour), RWC (%) relative water content, TGW (g) thousand-grain weight, GY (g/m^2^) grain yield, He hectoliter (kg/he), WSRC Water Solvent retention capacity, SCSRC Sodium carbonate Solvent retention capacity, SuSRC Sucrose Solvent retention capacity, LASRC Lactic acid Solvent retention capacity, MBSSRC Sodium Metabisulfite Solvent retention capacity, Cacl_2_SRC Chloride calcium Solvent retention capacity, SDSSRC Sodium dodecyl sulfate Solvent retention capacity, SDS+MBSSRC Sodium dodecyl sulfate + Sodium Metabisulfite Solvent retention capacity, EtSRC Ethanol Solvent retention capacity, GPI Gluten Performance Index.

### Synthetic or common wheats?

Significant genetic diversity was observed due to the differentiation of the genotype effect into common wheat and SHW-DL for all studied traits (S3 Table). The findings revealed that the RWC, TGW, GY, and all of the SRC traits (except WSRC) in SHW-DL were greater than common wheat under water stress conditions, while protein and RMT were greater than those in common wheat (Table 3). The traits including moisture, Zeleny, water absorbance, hardness, He, WSRC, and GPI showed no difference among genotypes of SHW-DL and common wheat under water stress conditions (Table 3). Under normal conditions, RWC, TGW, He, SuSRC, and SDS+MBSSRC in SHW-DL were greater than in common wheat, while protein, Zeleny, water absorbance, and RMT were higher in common wheat (Table 3).

**Table 3:** Comparison between synthetic (SHW) and common wheat using morphological, agronomic and quality traits during 2018–2019.

Traits	Normal		Stress	
	SHW	Common wheat	SHW	Common wheat
**Protein(%)**	16.39^b^	18.06^a^	17.65^b^	18.70 ^a^
**Moisture(%)**	8.45^a^	8.35^a^	8.34^a^	8.18 ^a^
**Zeleny(mL)**	59.15^b^	68.12^a^	60.05^a^	64.12 ^a^
**Water Absorbance(mL)**	66.86^b^	68.28^a^	67.01^a^	67.15 ^a^
**Hardness(%)**	64.70^a^	68.18^a^	63.91^a^	65.87 ^a^
**RMT(ml /100 gr flour)**	765.33^b^	864.38^a^	825.59 ^b^	890.25 ^a^
**RWC(%)**	76.85 ^a^	73.23 ^b^	60.51 ^a^	54.80 ^b^
**TGW(g)**	40.83^a^	35.99^b^	29.09 ^a^	25.94 ^b^
**GY(g/m** ^**2**^ **)**	1098.64 ^a^	1066.09 ^a^	859.94 ^a^	771.81 ^b^
**He(kg/he)**	83.80^a^	81.39^b^	76.21 ^a^	74.70 ^a^
**WSRC**	190.18^a^	193.26^a^	213.50 ^a^	207.49 ^a^
**SCSRC**	218.55^a^	221.65^a^	231.29 ^a^	224.10 ^b^
**SuSRC**	227.34 ^a^	217.24 ^b^	239.39 ^a^	231.36 ^b^
**LASRC**	203.21^a^	201.02^a^	215.55 ^a^	211.58 ^b^
**MBSSRC**	198.98 ^a^	195.59 ^a^	206.84 ^a^	197.55 ^b^
**Cacl2SRC**	222.30^a^	224.17^a^	234.09^a^	228.00^b^
**SDSSRC**	188.84^a^	186.36^a^	202.61^a^	197.38^b^
**SDS+MBSSRC**	192.19^a^	185.22^b^	206.27^a^	196.56^b^
**EtSRC**	190.85^a^	192.18^a^	209.60^a^	202.82^b^
**GPI**	45.60^a^	45.72^a^	45.49^b^	47.02^a^

RMT rapid mix test (ml/100 gr flour), RWC (%) relative water content, TGW (g) thousand-grain weight, GY (g/m^2^) grain yield, He hectoliter (kg/he), WSRC Water Solvent retention capacity, SCSRC Sodium carbonate Solvent retention capacity, SuSRC Sucrose Solvent retention capacity, LASRC Lactic acid Solvent retention capacity, MBSSRC Sodium Metabisulfite Solvent retention capacity, Cacl_2_SRC Chloride calcium Solvent retention capacity, SDSSRC Sodium dodecyl sulfate Solvent retention capacity, SDS+MBSSRC Sodium dodecyl sulfate + Sodium Metabisulfite Solvent retention capacity, EtSRC Ethanol Solvent retention capacity, GPI Gluten Performance Index.

### Association of traits and screening of genotypes

There were significant and negative correlations between grain yield and Pro, as well as between grain yield and zeleny, based on the phenotypic correlations among different traits under normal conditions (Table 4). In contrast, these correlations were not significant under water stress conditions. Furthermore, grain yield revealed significant positive correlations with TGW and He under water stress conditions which was not the case under normal conditions. Under both moisture conditions, there are significant and positive associations among SRC-related traits, except for MBSSRC with SDS+MBSSRC and CaCl_2_SRC under water stress conditions, and for EtSRC with CaCl_2_SRC and SDS+MBSSRC under both soil moisture conditions (S1 Fig). Protein had associations with WABS, Zeleny, hardness, and RMT, which were significantly positive under both water conditions (S1 Fig). Furthermore, positive and significant correlations were seen between zeleny and WABS, as well as between zeleny and hardness under both moisture environments (S1 Fig). Significant positive relationships were observed between water absorbance with RMT and hardness under both conditions. RMT also showed significant and positive correlations with WABS and hardness under both conditions (Table 4).

**Table 4:** Correlation coefficients among different quality traits and tolerance index (CSI) of SHW population evaluated under normal (above diagonal) and water-stressed (below diagonal) environments.

Traits	WSRC	SCSRC	SuSRC	LASRC	GPI	MBSSRC	Cacl_2_SRC	SDSSRC	SDS + MBSSRC	EtSRC	Pro	MOI	ZEL	WABS	HAR	RMT	TGW	He	GY
**WSRC**	1	0.73**	0.71**	0.76**	0.01 ^ns^	0.67**	0.73**	0.67**	0.65**	0.16 ^ns^	0.03 ^ns^	0.00 ^ns^	0.26 ^ns^	0.31*	0.16 ^ns^	0.07 ^ns^	0.11 ^ns^	0.20 ^ns^	0.19 ^ns^
**SCSRC**	0.59**	1	0.77**	0.77**	-0.23 ^ns^	0.78**	0.71**	0.67**	0.64**	0.19 ^ns^	0.27 ^ns^	0.09 ^ns^	0.33*	0.17 ^ns^	0.36**	0.16 ^ns^	0.21 ^ns^	0.30*	0.33*
**SuSRC**	0.69**	0.87**	1	0.67 **	-0.24 ^ns^	0.68 **	0.68**	0.64 **	0.64**	0.63 **	0.06 ^ns^	0.18 ^ns^	-0.13 ^ns^	0.12 ^ns^	0.19 ^ns^	0.23 *	-0.23 *	-0.25 *	0.04 ^ns^
**LASRC**	0.79**	0.78**	0.87**	1	0.35*	0.76 **	0.80**	0.67**	0.72**	0.65 **	0.01 ^ns^	-0.01 ^ns^	-0.18 ^ns^	0.11 ^ns^	0.21 *	0.19 ^ns^	-0.14^ns^	-0.28 **	0.06 ^ns^
**GPI**	0.41**	-0.08 ^ns^	0.10 ^ns^	0.50**	1	0.02 ^ns^	0.14 ^ns^	0.02 ^ns^	0.10 ^ns^	0.17 ^ns^	-0.06 ^ns^	-0.05 ^ns^	0.35*	0.54*	0.06 ^ns^	0.07 ^ns^	0.06 ^ns^	0.10 ^ns^	-0.03 ^ns^
**MBSSRC**	0.75**	0.82**	0.81 **	0.80 **	0.22 ^ns^	1	0.74**	0.71 **	0.74**	0.74 **	0.09 ^ns^	0.08 ^ns^	-0.15 ^ns^	0.16 ^ns^	0.24 *	0.29 **	-0.21 *	-0.30 **	0.01 ^ns^
**Cacl**_**2**_ **SRC**	0.66**	0.90**	0.90**	0.86**	0.11 ^ns^	0.11 ^ns^	1	0.69**	0.75**	0.19 ^ns^	0.07 ^ns^	-0.01 ^ns^	0.28*	0.32*	0.18 ^ns^	0.08 ^ns^	0.17 ^ns^	0.18 ^ns^	0.14 ^ns^
**SDSSRC**	0.66**	0.85**	0.80 **	0.75 **	0.11 ^ns^	0.77 **	0.85**	1	0.70**	0.75**	0.06 ^ns^	-0.00 ^ns^	-0.20 *	0.15 ^ns^	0.22 *	0.25 *	-0.20 *	-0.33 **	-0.04 ^ns^
**SDS+MBSSRC**	0.76**	0.88**	0.89**	0.88**	0.19 ^ns^	0.19 ^ns^	0.90**	0.87**	1	0.12 ^ns^	0.18 ^ns^	0.10 ^ns^	0.35*	0.26 ^ns^	0.16 ^ns^	0.17 ^ns^	0.20 ^ns^	0.37**	0.25 ^ns^
**EtSRC**	-0.01 ^ns^	0.09 ^ns^	0.71 **	0.66 **	-0.14 ^ns^	0.63 **	0.05 ^ns^	0.54 **	0.03 ^ns^	1	0.35**	-0.05 ^ns^	0.00 ^ns^	0.38 **	0.46**	0.52 **	-0.25 *	-0.23 *	-0.02 ^ns^
**Pro**	0.15 ^ns^	0.21 ^ns^	0.11 ^ns^	0.08 ^ns^	0.00 ^ns^	0.13 ^ns^	0.11 ^ns^	-0.01 ^ns^	0.17 ^ns^	0.32**	1	-0.26**	0.62**	0.64 **	0.61 **	0.87 **	-0.19 ^ns^	0.02 ^ns^	-0.33 **
**MOI**	0.08 ^ns^	0.22 ^ns^	-0.12 ^ns^	-0.23 *	0.01 ^ns^	-0.15 ^ns^	0.19 ^ns^	-0.09 ^ns^	0.17 ^ns^	-0.13 ^ns^	-0.22*	1	-0.14 ^ns^	-0.17^ns^	-0.23 *	-0.16 ^ns^	-0.18 ^ns^	-0.12 ^ns^	-0.04 ^ns^
**ZEL**	0.27 ^ns^	0.28*	-0.07 ^ns^	-0.03 ^ns^	0.16 ^ns^	-0.01 ^ns^	0.27 ^ns^	-0.11 ^ns^	0.31*	0.09 ^ns^	0.64**	-0.14 ^ns^	1	0.68**	0.58 **	0.27 **	0.01 ^ns^	0.10 ^ns^	-0.28 **
**WABS**	0.28*	0.12 ^ns^	0.17 ^ns^	0.22 *	0.29 ^ns^	0.16 ^ns^	0.21 ^ns^	0.12^ns^	0.27 ^ns^	0.34 **	0.61**	-0.23 *	0.68 **	1	0.96 **	0.61**	-0.08 ^ns^	0.11 ^ns^	-0.20*
**HAR**	0.03 ^ns^	0.13 ^ns^	0.22 *	0.28 **	-0.03 ^ns^	0.20 *	0.04 ^ns^	0.17 ^ns^	0.07 ^ns^	0.37 **	0.58 **	-0.31 **	0.57 **	0.97 **	1	0.65 **	-0.06 ^ns^	0.10 ^ns^	-0.17^ns^
**RMT**	0.11 ^ns^	0.07 ^ns^	0.23 *	0.20 *	0.06 ^ns^	0.20 *	0.03 ^ns^	0.10 ^ns^	0.11 ^ns^	0.39 **	0.87 **	-0.19 ^ns^	0.32 **	0.60 **	0.65**	1	-0.23*	0.00 ^ns^	-0.25 *
**TGW**	-0.04 ^ns^	0.04 ^ns^	-0.22 *	-0.20 *	0.00 ^ns^	-0.30 **	0.03 ^ns^	-0.25 *	0.04 ^ns^	-0.22 *	-0.05 ^ns^	-0.07 ^ns^	0.08 ^ns^	-0.02 ^ns^	-0.01 ^ns^	-0.09 ^ns^	1	0.18 ^ns^	0.17^ns^
**He**	0.08 ^ns^	0.07 ^ns^	-0.40*	-0.42 **	0.10 ^ns^	-0.47 **	0.03 ^ns^	-0.42*	0.15 ^ns^	-0.28 **	0.07 ^ns^	0.05 ^ns^	0.26 **	0.04 ^ns^	0.00 ^ns^	-0.03 ^ns^	0.51 **	1	0.11 ^ns^
**GY**	0.11 ^ns^	0.30*	-0.16 ^ns^	-0.13 ^ns^	-0.02 ^ns^	-0.22 *	0.22 ^ns^	-0.14 ^ns^	0.17 ^ns^	-0.01 ^ns^	-0.06 ^ns^	0.08 ^ns^	-0.03 ^ns^	0.00 ^ns^	0.02 ^ns^	-0.03 ^ns^	0.26 **	0.36 **	1
**CSI**	0.01 ^ns^	0.05 ^ns^	-0.15 ^ns^	-0.14 ^ns^	-0.16 ^ns^	-0.20 *	-0.05 ^ns^	-0.08 ^ns^	-0.02 ^ns^	-0.06 ^ns^	-0.25 *	0.06 ^ns^	-0.20 *	-0.13 ^ns^	-0.09 ^ns^	-0.19 ^ns^	0.25*	0.27 **	0.89 **

Solvent retention capacity, SCSRC Sodium Carbonate Solvent retention capacity, SuSRC Sucrose Solvent retention capacity, LASRC Lactic Acid Solvent retention capacity, GPI Gluten Performance Index, MBSSRC Sodium Metabisulfite Solvent retention capacity, Cacl_2_SRC Chloride calcium Solvent retention capacity, SDSSRC Sodium dodecyl sulfate Solvent retention capacity, SDS+MBSSRC Sodium dodecyl sulfate + Sodium Metabisulfite Solvent retention capacity, EtSRC Ethanol Solvent retention capacity, Pro Protein, MOI Moisture, ZEL Zeleny, WABS Water Absorbance, HAR Hardness, RMT rapid mix test (ml/100 gr flour), TGW (g) thousand-grain weight, He hectoliter (kg/he), GY (g/m^2^) grain yield, CSI Combination of significant index.

**Fig 1:**
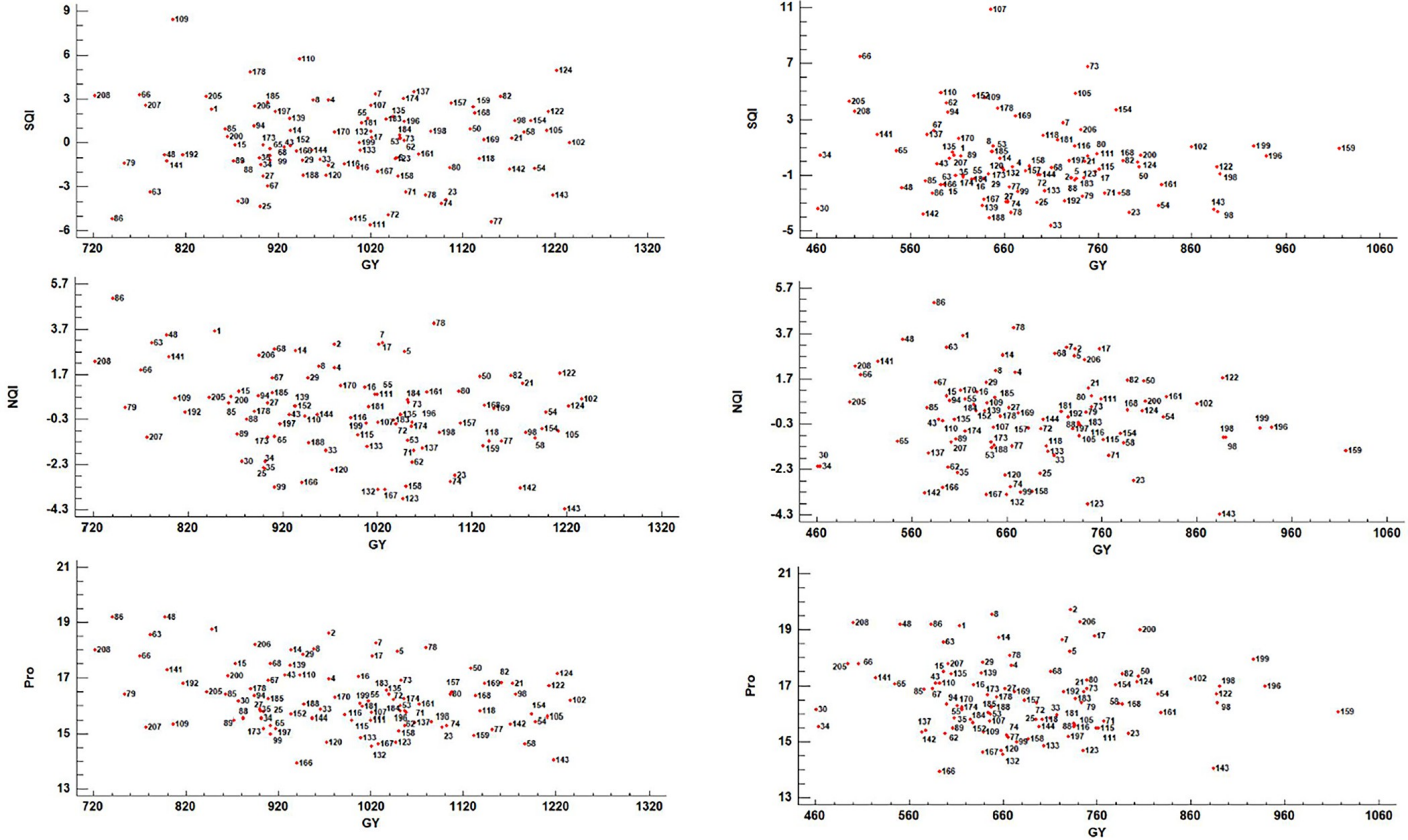
**a.** Biplot of GY with SQI (SRC quality index), NQI (NIR quality index) and Pro (protein content) for 91 synthetic hexaploid wheat and 8 common wheats under normal conditions (list of genotypes is presented in S2 Table). **b.** Biplot of GY with SQI (SRC quality index), NQI (NIR quality index) and Pro (protein content) for 91 synthetic hexaploid wheat and 8 common wheats under water stress conditions (list of genotypes is presented in S2 Table).

Means of essential quality traits in synthetic genotypes under well-irrigated and water-stressed environments are shown in S4 Table. The bi-plot for grain yield with SQI, NQI, and Pro was depicted in Fig 1A and 1B. According to the bi-plot, genotypes 21, 50, 54, 58, 82, 98, 102, 105, 122, 124, 154, 157, 159, 168, 169, and 198 had higher values for grain yield and quality under normal conditions (Fig 1A). For water stress conditions, genotypes 50, 82, 102, 122, 124, 154, 159, 161, 168, 196, 198, and 199 exhibited higher values for grain yield and quality (Fig 1B). Genotypes 21, 23, 50, 54, 74, 77, 80, 82, 98, 102, 105, 118, 122, 124, 142, 154, 157, 159, 168, 169, and 198 demonstrated higher grain quality under normal environments (Fig 1A). In contrast, genotypes 50, 54, 58, 82, 98, 102, 122, 124, 154, 159, 161, 168, 196, 198, and 199 showed higher grain quality under water stress environments (Fig 1B). Genotypes 8, 14, 15, 29, 43, 48, 63, 66, 67, 68, 86, 110, 139, and 141 revealed higher protein content with moderate grain yield under normal water conditions (Fig 1A). Genotypes 7, 8, 14, 15, 16, 17, 21, 29, 43, 48, 63, 65, 66, 68, 78, 80, 86, 110, 135, 139, and 141 showed higher protein with moderate grain yield under water-stressed environments (Fig 1B).

**Fig 2:**
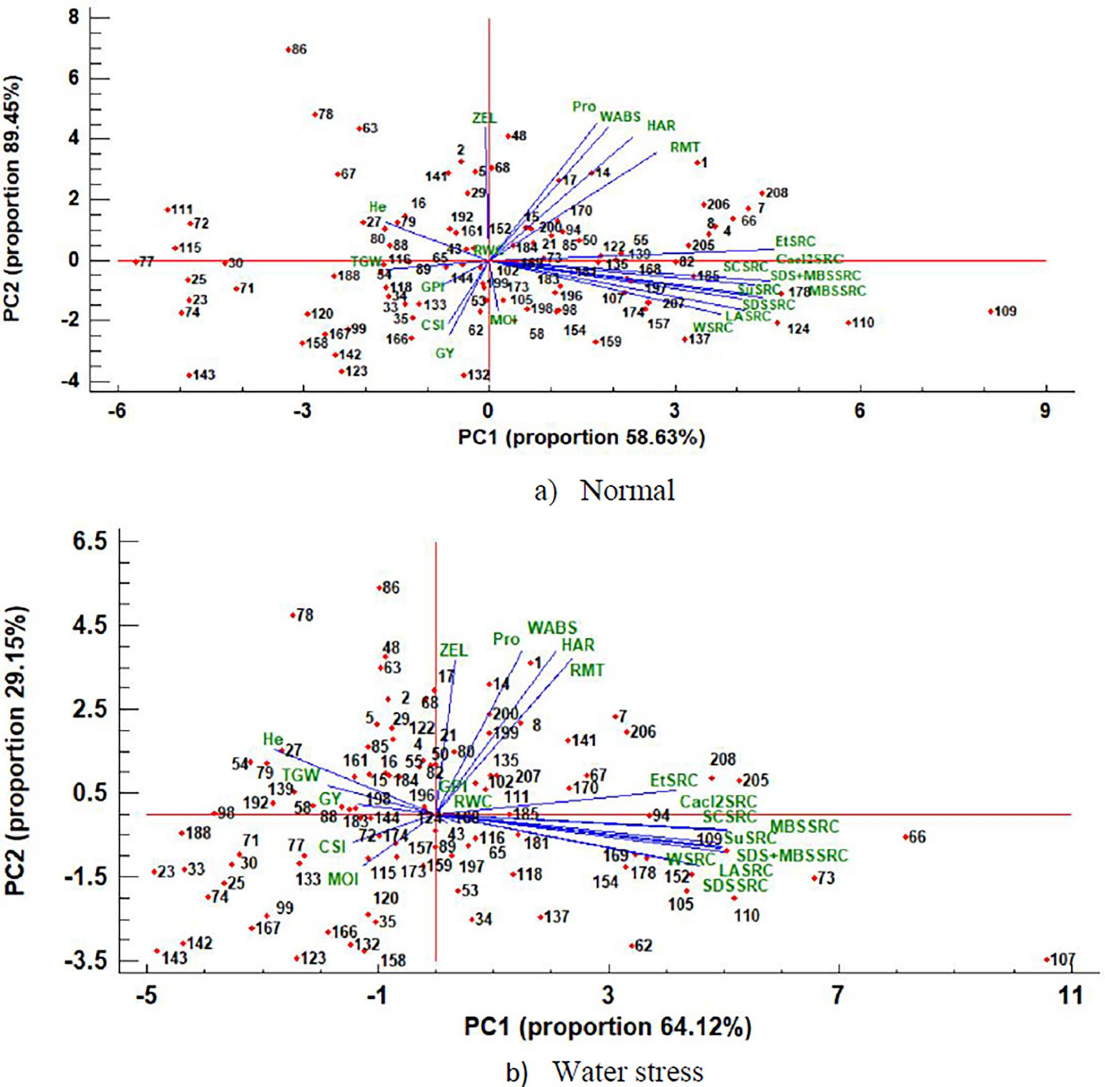
Principal component analysis of traits measured in the synthetic hexaploid wheats derived lines under normal (a) and water stress (b) conditions. Horizontal and vertical axes are the first and second principal components, respectively. WSRC Water Solvent retention capacity, SCSRC Sodium carbonate Solvent retention capacity, LASRC Lactic acid Solvent retention capacity, SuSRC Sucrose Solvent retention capacity, MBSSRC Sodium Metabisulfite Solvent retention capacity, CaCl2SRC Chloride calcium Solvent retention capacity, SDSSRC Sodium dodecyl sulfate Solvent retention capacity, SDS+MBSSRC Sodium Metabisulfite Sodium dodecyl sulfate + Solvent retention capacity, EtSRC Ethanol Solvent retention capacity, Pro Protein, MOI Moisture, ZEL Zeleny, WABS Water Absorbance, HAR Hardness, RMT rapid mix test (ml/100 gr flour), TGW (g) thousand-grain weight, RWC Relative water content, He hectoliter (kg/he), GY (g/m^2^) grain yield, CSI Combination of significant index. The Number of genotypes is according to the first column (Genotype code) in S2 Table.

Based on productivity, quality, and the indices of drought tolerance, a multivariate analysis (such as PCA) was conducted for normal and water stress environments to understand the correlation between genotypes and traits (Fig 2A and 2B). The first two components of PC explained 58.63% and 64.12% of the variation under water stress and normal environments, respectively. The PC demonstrated that the measured traits were distinctly into three groups, separately. The first group included traits related to SRC (WSRC, SCSRC, SuSRC, LASRC, Cacl_2_SRC, MBSSRC, SDS+MBSSRC, SDSSRC, and EtSRC), the second group contained grain yield and its components, and f the final group included quality-related traits associated with NIR. The results showed that SRC-related traits had a correlation which is strongly positive and were highly associated with high PC1 under both moisture conditions. In both moisture regimes, grain yield, its components, and the drought tolerance index showed a strong negative association with PC1. High PC2 and moderate PC1 represented the locations of NIR-related traits, which included WABS, RMT, Pro, ZEL, MOI, and HAR. The principal component analysis revealed that genotype 82, 107, 109, 110, 122, 124, 157, 168, 174, 178, 185, and 197 exhibited higher values of SRC-related traits under normal conditions. Genotypes 66, 73, 94, 105, 109, 110, 118, 152, 154, 168, 169, 178, 181, and 185 had higher values of quality traits (SRC-related traits) under water stress conditions. NIR-related traits correlated strongly positive with each other, and the genotypes including 14, 15, 17, 29, 48, 68, 94, 170, and 200 under normal conditions and 8, 14, 17, 21, 50, 80, 82, 135, 141, 199, and 200 displayed greater values of these traits under water stress conditions (Fig 2). SRC-related traits correlated highly with PC1; therefore, it may be referred to as an “SRC” factor (Fig 2A). Conversely, PC2 positively correlated with NIR-related traits, positively (Zeleny, protein, water absorbance, hardness, and RMT), which was designated as a “NIR” factor (Fig 2A). To achieve superior genotypes in terms of quality traits, the regions with high PC1 and PC2 have been selected, including 1, 4, 7, 8, 66, 205, 206, and 208.

## Discussion

The assessment of wheat end-use quality is an important goal for plant breeders to fulfil the demands for meals for the developing global population using several quality parameters. Due to drought being one of the significant production constraints in recent years, finding genotypes suited to different conditions is a necessity. Since wheat germplasm has experienced a genetic bottleneck throughout its evolution and plant breeding efforts, SHW-DL has been proposed as an alternative to widen the genetic variation related to common wheat. Untill now, the grain quality of SHW-DL and its tolerance to abiotic stresses have been overlooked. The present study assessed the main flour functional properties, grain yield, and indices of drought tolerance related to SHW and common wheat genotypes to introduce the best SHW-DL genotypes.

In this study, the observation of considerable genetic variations (high GCV and h^2^) for the studied traits unveiled the high capacity of SHW-DL genotypes to enhance these traits in breeding program. The categorization of h^2^ was high for major variables (except GY) according to the classification of Dabholkar (1992), especially under water stress conditions [21]. The finding was consistent with recent research that estimated high heritability for the major evaluated variables except for GY and ZEL under both water environments [16]. It was suggested that the high heritability of most traits emphasized the existence of key genes influencing them [16].

In the present study, the water deficit affected most of the evaluated traits by either increasing or decreasing them. As expected, the effect of water stress led to a 31% decrease in production in comparison with normal environments, in which a 36% decrease was reported under water stress conditions [22]. The yield reduction under water deficit could be attributed to stomatal conductance, lower water potential, photosynthetic rates, transpiration, dry matter accumulation, and distribution of the grain filling period [23]. Synthetic wheat can transfer metabolites from leaves and stems to the grain more rapidly than common wheat under drought stress [24]. The results might be proposed that this germplasm is suitable for selecting genotypes that are adaptable and appropriate for drought resistance due to various reactions to water stress observed in this germplasm.

The SRC method is a novel and distinctive approach to measuring flour functionality based on the expansion characteristics of polymerized flour constituent structures. We evaluated a panel of 99 wheat genotypes (mainly new advanced synthetics). The results showed a wide variation in the values of SRC-related traits, ranging from 129–317 (for WSRC) to 182–253 (for LASRC). This finding contrasts with the results of Guzman et al. (2015) who found the most significant variation in LASRC and the least in WSRC [19]. Zeleny varied between 24–107, which revealed that all the evaluated SHW-DL flours in this study could have a bread-making potential, according to Duyvejonck et al. (2011), owing to their Zeleny values exceeding 20 ml [25]. Measuring water absorption (WABS) is the primary responsibility of bakers to improve flour dough handling and to facilitate the preparation of bakery products [26]. There was a wide range of WABS, between 60 and 72, which aligns with the findings of Chavoushi et al. (2022) and Saeidnia et al. (2023) [15,16].

Comparison between SHW-DL and common wheat revealed that RWC, TGW, SuSRC, and SDS+MBSSRC were higher in SHW-DL under both conditions, while protein and RMT were higher in common wheat. Moreover, there was no difference in grain yield under normal conditions, but the importance of SHW-DL appeared under water stress conditions, where it exhibited higher grain yield. Therefore, the results demonstrated the superiority of SHW-DL under water stress compared to common wheat. The LASRC, SCSRC, SuSRC, and WSRC are directly related to the content of glutenin, the amount of damaged starch, available pentosan, and overall water-holding capacity, respectively [25,27]. Good gluten strength, high water absorption, damaged starch, and arabinoxylans are the properties of quality bread flour. In contrast, good flour typically has the lowest water absorption, damaged starch, strength of gluten, and arabinoxylans suitable for cookies. The results indicated that higher values of main solvents (LASRC, SCSRC, SuSRC, and WSRC) and supplemental solvents (MBSSRC, CaCl_2_SRC, SDS+MBSSRC, SDSSRC, and EtSRC) were attributed to SHW-DL under water stress conditions. Hence, SHW-DL exhibited higher glutenin content, amount of damaged starch, available pentosane, overall water holding capacity, and gluten strength (glutenin and gliadin strength) compared to common wheat. Since gliadin and glutenin (two important parameters for bread-baking performance in hard bread wheats contributed to dough viscosity and dough elasticity, respectively [28], SHW-DL genotypes were recommended for bread-making. Therefore, these findings suggest that the SRC has a robust capacity to differentiate the quality of wheat genotypes.

To find effective criteria for indirect selection, determining the interrelationship between traits is a prerequisite. One valuable way to improve complex traits (including yield and quality traits) is to evaluate the correlation of simple traits, linked to essential traits. Recently, it has become a challenge in agriculture to consistently select for grain yield and quality. Moreover, grain yield (the important trait) has low heritability in this study, which caused in a lower probability of improving production. In the present study, quality-related traits could be a good choice for selecting the genotypes more effectively due to easy measurements and their correlation with complex traits such as grain yield [29]. The correlation coefficients indicated a significantly positive association between SCSRC and grain yield under both moisture conditions, revealing that producing greater amounts of damaged starch could help in breeding for high-yielding varieties. Decreasing the grain moisture makes the grain harder and produces more damaged starch. Grinding harder grain requires more pressure, leading to greater production of damaged starch in the flour [27,16]. More damaged starch increases flour water absorption during bread-making, resulting in better bread quality. Grain yield (GY) was correlated significantly negative with MBSSRC under water stress conditions. Overall, gluten strength was assessed using the MBSSRC solution [10]. In this regard, it is expected that high-yield genotypes under water stress conditions will exhibit low gluten strength. Furthermore, there were negative and significant correlations between protein and ZEL with grain yield under normal conditions. Moreover, the significant and positive relationship between ZEL with HAR and WABS under both moisture environments proposed that selecting for high ZEL and Pro may obliquely enhance the quality of wheat grain for bread-making. These outcomes are in accordance with those recently published in different species of wheat and triticale [15,16]. The significant and positive correlations of Pro with WABS, HAR, ZEL, and RMT demonstrated that higher grain protein leads to improved quality and hardiness. Hardness of grain is a key factor in wheat products, with hard grain primarily used for making bread. [30]. Conversely, the positive relationships between SuSRC and RMT as well as HAR indicated that the tougher genotypes possessed greater levels of pentosans, making them ideal for bread production. Overall, soft wheat items and hard wheat items require flour with low and high moisture absorption, respectively. The findings indicated that EtSRC had strong and positive correlations with Pro, WABS, HAR, RMT, and He suggesting that any increases in protein, water absorption, grain hardness, and grain size could be linked to a greater level of flour gliadin. In the PCA graph, all of the SRC traits were grouped closely, which is in accordance with a recent study that also showed significant relationships between these SRC variables [16].

Genotypes were identified using quality-related traits (SRC and NIR), productivity, and protein. Genotypes 82, 102, 122, 124, 154, 168, and 169 revealed a higher rank due to quality and grain yield. Genotypes 66, 73, 94, 109, 110, 152, 169, and 178 were identified as having high flour quality, especially SRCs under water stress conditions. The SHW-DLs can also be directly or indirectly used as superior genotypes in the process of wheat breeding process.

## Conclusions

In conclusion, observing more genetic diversity for all measured traits revealed that the examined SHW-DL population possesses functional genetic diversity for quality traits, yield, and drought tolerance. These results may be useful for future program of wheat breeding aimed at addressing challenges posed by global ecological shifts, particularly in arid and semi-arid regions. The superiority of SHW-DL was attributed to higher seed yield, glutenin content, amount of damaged starch levels, available pentosane, overall water holding capacity, and gluten strength under water stress, making it more suitable for bread-baking compared to common wheat. Moreover, these results showed that the SRC values could differentiate and highlight the quality of SHW-DL genotypes. The high yield associated with SHW-DL was linked to elevated damaged starch levels, greater water absorption, hardness, and lower gluten strength, ZEL, and protein in water-stress environments. These results hold important consequences for improving the quality of wheat varieties by utilizing SHW as genetic resources overcome the genetic bottleneck. Superior genotypes were identified for future studies.

## Supporting information

S1 Table Family pedigree of 91 synthetic hexaploid wheats used in this study. (DOCX)

S2 Table List and pedigree of synthetic derived hexaploid wheat lines along with 8 common wheats. Genotype identification (GID), introduction number (INTRID), Cross identification (CID), Synthetic Identification (SID), pedigree and synthetic degree are provided. The diploid D-genome donor (Aegilops tauschii) accession used in the synthetic cross is bolded and the tetraploid AB genome donor accession (Triticum turgidum) used in the cross is underlined.
(DOCX)

S3 Table Analysis of variance for quality and agronomic traits tested under well-watered and drought stressed conditions in synthetic hexaploid wheats during two years. (DOCX)

S4 Table Means of important quality traits in synthetic wheat genotypes under well-irrigated and drought stressed conditions during 2018–2019. (DOCX)

S1 Fig Correlation coefficients among different quality traits and tolerance index (CSI) of SHW population evaluated normal (a) and water stress (b) conditions. WSRC Water Solvent retention capacity, SCSRC Sodium carbonate Solvent retention capacity, LASRC Lactic acid Solvent retention capacity, SuSRC Sucrose Solvent retention capacity, MBSSRC Sodium Metabisulfite Solvent retention capacity, CaCl2SRC Chloride calcium Solvent retention capacity, SDSSRC Sodium dodecyl sulfate Solvent retention capacity, SDS+MBSSRC Sodium Metabisulfite Sodium dodecyl sulfate + Solvent retention capacity, EtSRC Ethanol Solvent retention capacity, PRO Protein, MOI Moisture, ZEL Zeleny, WABS Water Absorbance, HAR Hardness, RMT rapid mix test (ml/100 gr flour), TGW (g) thousand-grain weight, RWC Relative water content, He hectoliter (kg/he), GY (g/m2) grain yield, CSI Combination of significant index.
(DOCX)
