# Supplementary material for: Synthetic wheat as a new source of flour quality under drought conditions: Associations with solvent retention capacity
Source: PLoS One. 2025 Feb 6;20(2):e0316945. doi: 10.1371/journal.pone.0316945 (PMC11801611; doi:10.1371/journal.pone.0316945)
Supplement: S1 Table — (DOCX) [file pone.0316945.s001.docx]

| **S1 Table.** **Family pedigree of 91 synthetic hexaploid wheats used in this study** | | | |
| --- | --- | --- | --- |
| ***T. turgidum* donor** | ***Ae. tauschii* donor** | **No. of lines** | **Primary Synthetic Hexaploid parent** |
| CERCETA | *Ae. searsii* (34D) | 2 | CERCETA/*Ae. searsii* (34D) |
| ALTAR 84 | *Ae. squarrosa* (193) | 1 | ALTAR 84/*Ae. squarrosa* (193) |
| CROC_1 | *Ae. squarrosa* (205) | 9 | CROC_1/ *Ae. squarrosa* (205) |
| Gediz75/3/Goose//Albatros:Dr/Crane | *Ae. squarrosa* (208) | 2 | Gediz75/3/Goose//Albatros:Dr/Crane/*Ae. squarrosa* (208) |
| CROC_1 | *Ae. squarrosa* (213) | 9 | CROC_1/ *Ae. squarrosa* (213) |
| DVERD_2 | *Ae. squarrosa* (214) | 7 | DVERD_2/ *Ae. squarrosa* (214) |
| ALTAR 84 | *Ae. squarrosa* (224) | 1 | ALTAR 84/ *Ae. squarrosa* (224) |
| CROC_1 | *Ae. squarrosa* (224) | 43 | CROC_1/ *Ae. squarrosa* (224) |
| SORA | *Ae. squarrosa* (323) | 5 | SORA/ *Ae. squarrosa* (323) |
| STERNA:DR | *Ae. squarrosa* (358) | 6 | STERNA:DR/ *Ae. squarrosa* (358) |
| YARMUK | *Ae. squarrosa* (434) | 3 | YARMUK/ *Ae. squarrosa* (434) |
| DECOY 1 | *Ae. squarrosa* (458) | 3 | DECOY 1/ *Ae. squarrosa* (458) |
| CROC_1 | *Ae. squarrosa* (662) | 18 | CROC_1/ *Ae. squarrosa* (662) |
| CRANE | *Ae. squarrosa* (895) | 1 | CRANE/ *Ae. squarrosa* (895) |
| ALTAR 84 | *Ae. squarrosa* | 3 | ALTAR 84/ *Ae. Squarrosa* |
| CHEN | *Ae. squarrosa* | 2 | CHEN/ *Ae. Squarrosa* |
| ALTAR 84 | *Ae. squarrosa* (Taus) | 26 | ALTAR 84/ *Ae. squarrosa* (Taus) |
| Cando/R143//Ente/Mexicali_2 | *Ae. squarrosa* (Taus) | 18 | Cando/R143//Ente/Mexicali_2/ *Ae. squarrosa* (Taus) |
| CHEN | *Ae. squarrosa* (Taus) | 10 | CHEN/ *Ae. squarrosa* (Taus) |
| ALTAR84 | *Ae. squarrosa* (219) | 3 | ALTAR84/ *Ae. squarrosa* (219) |
| DECOY 1 | *Ae. squarrosa* (510) | 3 | DECOY 1/ *Ae. squarrosa* (510) |
| CROC_1 | *Ae. squarrosa* (879) | 5 | CROC_1/ *Ae. squarrosa* (879) |
| ALTAR84 | *Ae. squarrosa* (221) | 4 | ALTAR84/ *Ae. squarrosa* (221) |
