# Supplementary material for: Synthetic wheat as a new source of flour quality under drought conditions: Associations with solvent retention capacity
Source: PLoS One. 2025 Feb 6;20(2):e0316945. doi: 10.1371/journal.pone.0316945 (PMC11801611; doi:10.1371/journal.pone.0316945)
Supplement: S2 Table — Genotype identification (GID), introduction number (INTRID), Cross identification (CID), Synthetic Identification (SID), pedigree and synthetic degree are provided. The diploid D-genome donor (Aegilops tauschii) accession used in the synthetic cross is bolded and the tetraploid AB genome donor accession (Triticum turgidum) used in the cross is underlined. (DOCX) [file pone.0316945.s002.docx]

| **S2 Table. List and pedigree of synthetic derived hexaploid wheat lines along with 8 common wheats. Genotype identification (GID), introduction number (INTRID), Cross identification (CID), Synthetic Identification (SID), pedigree and synthetic degree are provided. The diploid D-genome donor (*Aegilops tauschii*) accession used in the synthetic cross is bolded and the tetraploid AB genome donor accession (*Triticum turgidum*) used in the cross is underlined.** | | | | | | |
| --- | --- | --- | --- | --- | --- | --- |
| **Genotype code** | **GID** | **INTRID** | **CID** | **SID** | **Synthetic degree** | **Pedigree** |
| **Common wheat** | |  |  |  |  |  |
| 1 | | AAC Scotia | - | - | - | Quantum/AC Walton |
| 2 | | Carberry | - | - | - | Alsen/Superb |
| 4 | | Hoffman | - | - | - | Flickers/Ankra//AC Brio |
| 5 | | Norwell | - | - | - | - |
| **Synthetic wheat** | |  |  |  |  |  |
| 7 | 2454874 | BW31682 | 72683 | 805 | 2^nd^ | Altar84/***Ae.Squarrosa*(219)**//SeriM 82 |
| 8 | 2454873 | BW31683 | 72683 | 806 | 2^nd^ | Altar84/***Ae.Squarrosa*(219)**//SeriM 82 |
| 14 | 2454969 | BW31694 | 72744 | 378 | 2^nd^ | Dverd_2/***Ae.Squarrosa*(214)**//OpataM85 |
| 15 | 2454968 | BW31695 | 72744 | 379 | 2^nd^ | Dverd_2/***Ae.Squarrosa*(214)**//OpataM85 |
| 16 | 2454967 | BW31696 | 72744 | 380 | 2^nd^ | Dverd_2/***Ae.Squarrosa*(214)**//OpataM85 |
| 17 | 2454966 | BW31697 | 72744 | 381 | 2^nd^ | Dverd_2/***Ae.Squarrosa*(214)**//OpataM85 |
| 21 | 2479186 | BW31782 | 167256 | 309 | 2^nd^ | Bacanora T88//Sora/***Ae.Squarrosa*** **(323)** |
| 23 | 1874471 | BW31784 | 167282 | 572 | 2^nd^ | Bacanora T88//Croc_1/***Ae.Squarrosa*** **(662)** |
| 25 | 1874482 | BW31786 | 167282 | 568 | 2^nd^ | Bacanora T88//Croc_1/***Ae.Squarrosa*** **(662)** |
| 27 | 1874477 | BW31788 | 167282 | 570 | 2^nd^ | Bacanora T88//Croc_1/***Ae.Squarrosa*** **(662)** |
| 29 | 1874460 | BW31790 | 167282 | 577 | 2^nd^ | Bacanora T88//Croc_1/***Ae.Squarrosa*** **(662)** |
| 30 | 1874466 | BW31791 | 167282 | 574 | 2^nd^ | Bacanora T88//Croc_1/***Ae.Squarrosa*** **(662)** |
| 33 | 1874454 | BW31794 | 167282 | 579 | 2^nd^ | Bacanora T88//Croc_1/***Ae.Squarrosa*** **(662)** |
| 34 | 1874446 | BW31796 | 167282 | 582 | 2^nd^ | Bacanora T88//Croc_1/***Ae.Squarrosa*** **(662)** |
| 35 | 1874426 | BW31797 | 167282 | 590 | 2^nd^ | Bacanora T88//Croc_1/***Ae.Squarrosa*** **(662)** |
| 43 | 1877008 | BW31806 | 167357 | 95 | 2^nd^ | Opata M85//Decoy 1/***Ae.Squarrosa*** **(510)** |
| 48 | 1877986 | BW31812 | 167384 | 168 | 2^nd^ | Opata M85//Croc 1/***Ae.Squarrosa*** **(879)** |
| 50 | 1118891 | BW32103 | 72726 | 530 | 2^nd^ | Croc 1/***Ae.Squarrosa*** **(224)**//Opata M85 |
| 53 | 2454892 | BW32107 | 72726 | 631 | 2^nd^ | Croc 1/***Ae.Squarrosa*** **(224)**//Opata M85 |
| 54 | 1403557 | BW32114 | 101714 | 72 | 4^th^ | Croc 1/***Ae.Squarrosa*** **(205)**//Jupateco F73/Bluejay/3/Super Kauz/4/K |
| 55 | 2453300 | BW32132 | 58520 | 34 | 2^nd^ | Chen/***Ae.Squarrosa***//2*Opata M85 |
| 58 | 1118888 | BW33631 | 72726 | 531 | 2^nd^ | Croc 1/***Ae.Squarrosa*** **(224)**//Opata M85 |
| 62 | 2478023 | BW33662 | 158324 | 43 | 3^rd^ | Pastor//Sitella/Mochis73/3/Chen/***Aegilops Squarrosa*** **(*Taus*)**//Bacanora T88 |
| 63 | 2478033 | BW33665 | 158325 | 44 | 3^rd^ | Pastor/3/Munia//Chen/Altar 84/5/Cando/R143//Ente/Mexicali 2/3/***Aegilops Squarrosa*** **(*Taus*)**/4/Weaver |
| 65 | 3616956 | BW33667 | 158452 | 132 | 3^rd^ | Filin/Irena/5/Cando/R143//Ente/Mexicali 2/3/***Aegilops Squarrosa*** **(*Taus*)**/4/Weaver |
| 66 | 3567694 | BW33670 | 158452 | 131 | 3^rd^ | Filin/Irena/5/Cando/R143//Ente/Mexicali 2/3/***Aegilops Squarrosa*** **(*Taus*)**/4/Weaver |
| 67 | 3632351 | BW33680 | 255660 | 97 | 3^rd^ | Chbia/5/Cando/R143//Ente/Mexicali 2/3/***Aegilops Squarrosa*** **(*Taus*)**/4/Weaver |
| 68 | 3577944 | BW33685 | 206381 | 227 | 3^rd^ | Croc 1/***Ae.Squarrosa*** **(213)**//Papago M86/3/CMH81.38/2*Kauz |
| 71 | 3628190 | BW33688 | 206499 | 26 | 2^nd^ | Altar 84/***Ae.Squarrosa*** **(219)**//Attila |
| 72 | 3567695 | BW33689 | 207697 | 27 | 3^rd^ | Cando/R143//Ente/Mexicali 2/3/***Aegilops Squarrosa*** **(*Taus*)**/4/Ocoroni F86/5/Pastor |
| 73 | 3628930 | BW33691 | 207963 | 31 | 3^rd^ | Munia/3/Ruff/Flamingo Dr//Yavaros79/4/Chen/***Aegilops Squarrosa (Taus)***//Bacanora T 88 |
| 74 | 3628949 | BW33693 | 207970 | 130 | 3^rd^ | Hoopoe/Tanager//Veery/3/2*Papago M86/4/Chen/***Aegilops Squarrosa (Taus)***//Bacanora T 88 |
| 77 | 3631236 | BW33728 | 210539 | 129 | 3^rd^ | Croc 1/***Ae.Squarrosa*** **(224)**//Opata M85/Kauz*2/Bobwhite//Kauz/4/NL 683 |
| 78 | 3631235 | BW33729 | 210539 | 130 | 3^rd^ | Croc 1/***Ae.Squarrosa*** **(224)**//Opata M85/Kauz*2/Bobwhite//Kauz/4/NL 683 |
| 79 | 3631234 | BW33731 | 210539 | 131 | 3^rd^ | Croc 1/***Ae.Squarrosa*** **(224)**//Opata M85/Kauz*2/Bobwhite//Kauz/4/NL 683 |
| 80 | 3621025 | BW33746 | 167276 | 215 | 2^nd^ | Bacanora T88//Cerceta/***Ae.Searsii*** **(34D)** |
| 82 | 3616097 | BW33757 | 152383 | 95 | 3^rd^ | Altar 84/***Ae.Squarrosa* (224)**/Cucurpe S86/3/PI 610755 |
| 85 | 3584454 | BW33952 | 135084 | 149 | 3^rd^ | Croc 1/***Ae.Squarrosa*** **(205)**//Borlaug M95/3/2*Milan |
| 86 | 2478018 | BW33977 | 158324 | 48 | 3^rd^ | Pastor//Sitella/Mochis73/3/Chen/***Aegilops Squarrosa*** **(*Taus*)**//Bacanora T88 |
| 88 | 2478027 | BW35684 | 158325 | 50 | 3^rd^ | Pastor/3/Munia//Chen/Altar 84/5/Cando/R143//Ente/Mexicali_2/3/***Aegilops Squarrosa*** **(*Taus*)**/4/Weaver |
| 89 | 3855011 | BW35697 | 279807 | 61 | 3^rd^ | Croc 1/***Ae.Squarrosa*** **(224)**//Opata M85/3/Pastor |
| 94 | 3616959 | BW36378 | 158452 | 134 | 3^rd^ | Filin/Irena/5/Cando/R143//Ente/Mexicali 2/3/***Aegilops Squarrosa*** **(*Taus*)**/4/Weaver |
| 98 | 3864981 | BW36536 | 304079 | 109 | 3^rd^ | Caskor/3/Croc_1/***Ae. Squarrosa*(224)**//Opata M85 |
| 99 | 3827947 | BW36542 | 279807 | 56 | 3^rd^ | Croc_1/***Ae. Squarrosa*(224)**//Opata M85/3/Pastor |
| 102 | 3855902 | BW36555 | 280805 | 59 | 4^th^ | Milan/Kauz/5/Cando/R143//Ente/Mexicali_2/3/***Aegilops Squarrosa*** **(*Taus*)**/4/Weaver/6/Tobari F66/Era//Tobari F66/Ciano F67/3/Pollo/4/Veery#5/5/Kauz |
| 105 | 3888537 | BW36562 | 334948 | 301 | 3^rd^ | Pastor/3/Altar 84/***Aegilops Squarrosa*** **(*Taus*)**//Opata M85 |
| 107 | 3888320 | BW36564 | 334948 | 313 | 3^rd^ | Pastor/3/Altar 84/***Aegilops Squarrosa*** **(*Taus*)**//Opata M85 |
| 109 | 4315350 | BW36568 | 331793 | 57 | 3^rd^ | Altar 84/***Ae.Squarrosa*** **(221)**//Pastor/3/Pastor |
| 110 | 2489102 | BW36874 | 255660 | 81 | 3^rd^ | Chibia/5/Cando/R143//Ente/Mexicali 2/3/***Aegilops Squarrosa*** **(*Taus*)**/4/Weaver |
| 111 | 2489138 | BW36875 | 255700 | 32 | 3^rd^ | Capeiti 8/5/Gediz 73/3/Goose//Albatros:Dr/Crane/4/***Ae. Squarrosa*** **(208)**/5/Hahn/2*Weaver |
| 115 | 3865440 | BW36880 | 304415 | 20 | 2^nd^ | Croc 1/***Ae.Squarrosa*** **(205)**//Milan//Kauz |
| 116 | 3865439 | BW36881 | 304415 | 21 | 2^nd^ | Croc 1/***Ae.Squarrosa*** **(205)**//Milan//Kauz |
| 118 | 3865437 | BW36883 | 304415 | 23 | 2^nd^ | Croc 1/***Ae.Squarrosa*** **(205)**//Milan//Kauz |
| 120 | 3855017 | BW36900 | 279807 | 64 | 3^rd^ | Croc 1/***Ae.Squarrosa*** **(224)**//Opata M85/3/Pastor |
| 122 | 3855013 | BW36902 | 279807 | 68 | 3^rd^ | Croc 1/***Ae.Squarrosa*** **(224)**//Opata M85/3/Pastor |
| 123 | 3855012 | BW36903 | 279807 | 69 | 3^rd^ | Croc 1/***Ae.Squarrosa*** **(224)**//Opata M85/3/Pastor |
| 124 | 3855034 | BW36904 | 279810 | 59 | 3^rd^ | Croc 1/***Ae.Squarrosa*** **(224)**//Opata M85/3/Pastor |
| 132 | 3888359 | BW36953 | 334948 | 294 | 3^rd^ | Pastor/3/Altar 84/***Aegilops Squarrosa*** **(*Taus*)**//Opata M85 |
| 133 | 3888358 | BW36954 | 334948 | 295 | 3^rd^ | Pastor/3/Altar 84/***Aegilops Squarrosa*** **(*Taus*)**//Opata M85 |
| 135 | 3888349 | BW36956 | 334948 | 264 | 3^rd^ | Pastor/3/Altar 84/***Aegilops Squarrosa*** **(*Taus*)**//Opata M85 |
| 137 | 4340793 | BW36965 | 331793 | 56 | 3^rd^ | Altar 84/***Ae. Squarrosa*** **(221)**//Pastor/3/Pastor |
| 139 | 2489095 | BW36981 | 255660 | 88 | 3^rd^ | Chibia/5/Cando/R143//Ente/Mexicali 2/3/***Aegilops Squarrosa*** **(*Taus*)**/4/Weaver |
| 141 | 3888399 | BW36984 | 334948 | 517 | 3^rd^ | Pastor/3/Altar 84/***Aegilops Squarrosa*** **(*Taus*)**//Opata M85 |
| 142 | 3833280 | BW37454 | 72726 | 797 | 2^nd^ | Croc 1/***Ae.Squarrosa*** **(224)**//Opata M85 |
| 143 | 4563458 | BW37461 | 303992 | 57 | 3^rd^ | Cando/R143//Ente/Mexicali 2/3/***Aegilops Squarrosa*** **(*Taus*)**/4/Weaver/5/Pastor |
| 144 | 4093487 | BW37467 | 304079 | 120 | 3^rd^ | Caskor/3/Croc_1/***Ae. Squarrosa*(224)**//Opata M85 |
| 152 | 4773725 | BW37606 | 365653 | 51 | 3^rd^ | Altar 84/***Aegilops Squarrosa*** **(*Taus*)**//Ocoroni F86/3/Veery/Marcos Juarez Inta/2*Tui |
| 154 | 3832784 | BW37608 | 58520 | 92 | 2^nd^ | Chen/***Ae.Squarrosa***//2*Opata M85 |
| 157 | 4563455 | BW37683 | 303992 | 60 | 3^rd^ | Cando/R143//Ente/Mexicali 2/3/***Aegilops Squarrosa*** **(*Taus*)**/4/Weaver/5/Pastor |
| 158 | 4062607 | BW37698 | 334948 | 1102 | 3^rd^ | Pastor/3/Altar 84/***Aegilops Squarrosa*** **(*Taus*)**//Opata M852 |
| 159 | 4577785 | BW37701 | 342438 | 56 | 3^rd^ | SuperKauz/Pastor/3/Croc_1/***Ae. Squarrosa*(224)**//Opata M85 |
| 161 | 4753157 | BW37707 | 342488 | 59 | 3^rd^ | Kaby/Baviacora M92/3/Croc 1/***Ae.Squarrosa*** **(224)**//Opata M85 |
| 166 | 4569078 | BW37857 | 334948 | 1095 | 3^rd^ | Pastor/3/Altar 84/***Aegilops Squarrosa*** **(*Taus*)**//Opata M852 |
| 167 | 4577760 | BW37864 | 342432 | 61 | 3^rd^ | SuperKauz/Baviacora M92/3/Croc_1/***Ae. Squarrosa*(224)**//Opata M85 |
| 168 | 4753160 | BW37867 | 342488 | 56 | 3^rd^ | Kaby/Baviacora M92/3/Croc 1/***Ae.Squarrosa*** **(224)**//Opata M85 |
| 169 | 4753159 | BW37868 | 342488 | 57 | 3^rd^ | Kaby/Baviacora M92/3/Croc 1/***Ae.Squarrosa*** **(224)**//Opata M85 |
| 170 | 4753158 | BW37869 | 342488 | 58 | 3^rd^ | Kaby/Baviacora M92/3/Croc 1/***Ae.Squarrosa*** **(224)**//Opata M85 |
| 173 | 4883007 | BW39383 | 363192 | 46 | 3^rd^ | Croc 1/***Ae.Squarrosa*** **(224)**//Opata M85/3/Altar 84/ ***Aegilops squarrosa (Taus)***//Opata M85/4/Pastor |
| 174 | 4883006 | BW39384 | 363192 | 49 | 3^rd^ | Croc 1/***Ae.Squarrosa*** **(224)**//Opata M85/3/Altar 84/ ***Aegilops squarrosa (Taus)***//Opata M85/4/Pastor |
| 178 | 4885603 | BW39413 | 373362 | 59 | 3^rd^ | Sterna:Dr/***Ae. Squarrosa*** **(358)**/3/Maioral/Buckbuck//Veery#7/4/Pastor |
| 181 | 4883372 | BW39450 | 365653 | 59 | 5^th^ | Altar 84/***Aegilops Squarrosa*** **(*Taus*)**//Ocoroni F86/3/Veery/Marcos Juarez Inta//2*Tui |
| 183 | 4883370 | BW39452 | 365653 | 61 | 5^th^ | Altar 84/***Aegilops Squarrosa*** **(*Taus*)**//Ocoroni F86/3/Veery/Marcos Juarez Inta//2*Tui |
| 184 | 4883369 | BW39453 | 365653 | 62 | 5^th^ | Altar 84/***Aegilops Squarrosa*** **(*Taus*)**//Ocoroni F86/3/Veery/Marcos Juarez Inta//2*Tui |
| 185 | 4883367 | BW39454 | 365653 | 64 | 5^th^ | Altar 84/***Aegilops Squarrosa*** **(*Taus*)**//Ocoroni F86/3/Veery/Marcos Juarez Inta//2*Tui |
| 188 | 4878715 | BW39478 | 342852 | 81 | 2^nd^ | Sterna:Dr/***Ae. Squarrosa*** **(358)**/4/Ures T81//Buckbuck//Pavon F76/3/Kauz/5/Ures T81/Junco/Kauz |
| 192 | 4750129 | BW39485 | 335446 | 82 | 3^rd^ | Croc 1/***Ae.Squarrosa*** **(213)**//Papago M86/3/Baviacora M92 |
| 196 | 4886016 | BW39489 | 378807 | 61 | 2^nd^ | Decoy 1/***Ae.Squarrosa*** **(458)**/3/Kauz/Gygis/Kauz |
| 197 | 4886015 | BW39490 | 378807 | 63 | 2^nd^ | Decoy 1/***Ae.Squarrosa*** **(458)**/3/Kauz/Gygis/Kauz |
| 198 | 4886014 | BW39491 | 378807 | 67 | 2^nd^ | Decoy 1/***Ae.Squarrosa*** **(458)**/3/Kauz/Gygis/Kauz |
| 199 | 6174895 | BW49397 | 520259 | 21 | 4^th^ | Altar 84/***Ae. Squarrosa*** **(221)**//3*Borlaug M95/3/Ures T81/Junco//Kauz/4/Weebilli/5/Mutus |
| 200 | 6174901 | BW49399 | 520259 | 27 | 4^th^ | Altar 84/***Ae. Squarrosa*** **(221)**//3*Borlaug M95/3/Ures T81/Junco//Kauz/4/Weebilli/5/Mutus |
| **Common wheat** | | **Name of wheat** | |  |  |  |
| 205 |  | Pishtaz | - | - | - |  |
| 206 |  | Roshan | - | - | - |  |
| 207 |  | Kavir | - | - | - |  |
| 208 |  | Ghods | - | - | - |  |
