# Supplementary material for: Synthetic wheat as a new source of flour quality under drought conditions: Associations with solvent retention capacity
Source: PLoS One. 2025 Feb 6;20(2):e0316945. doi: 10.1371/journal.pone.0316945 (PMC11801611; doi:10.1371/journal.pone.0316945)
Supplement: S3 Table — (DOCX) [file pone.0316945.s003.docx]

| \| **S3 Table. Analysis of variance for quality and agronomic traits tested under well-watered and drought stressed conditions in synthetic hexaploid wheats during two years** \| \| \| \| \| \| \| \| \| \| \| \| \| --- \| --- \| --- \| --- \| --- \| --- \| --- \| --- \| --- \| --- \| --- \| --- \| \| **SV** \| **df** \| **Mean Square** \| \| \| \| \| \| \| \| \| \| \| **Protein** \| **Moisture** \| **Zeleny** \| **Water Absorbance** \| **Hardness** \| **RMT** \| **RWC** \| **TGW** \| **GY** \| **He** \| \| **Irrigation (I)** \| 1 \| 77.43^**^ \| 0.73 ^ns^ \| 2.16 ^ns^ \| 0.07 ^ns^ \| 57.00 ^ns^ \| 171551.79 ^**^ \| 119077.63^**^ \| 51527.47^**^ \| 36567453.03 ^**^ \| 21465.70** \| \| **Year (Y)** \| 1 \| 1.83 ^ns^ \| 52.26^**^ \| 23698.28 ^**^ \| 55.97 ^**^ \| 4473.21^**^ \| 97987.61 ^*^ \| 6350.72 ^ns^ \| 53.25 ^ns^ \| 72333259.81^**^ \| 890.33 ^ns^ \| \| **Y * I** \| 1 \| 12.49^*^ \| 0.01 ^ns^ \| 212.16 ^ns^ \| 0.88 ^ns^ \| 26.46 ^ns^ \| 10573.75 ^ns^ \| 15154.62 ^ns^ \| 3888.09^**^ \| 570439.08 ^ns^ \| 6.87 ^ns^ \| \| **Rep (Y * I)** \| 8 \| 1.01 ^ns^ \| 1.19^**^ \| 172.70 ^*^ \| 5.11^*^ \| 50.82 ^**^ \| 5517.58 ^*^ \| 3929.07^**^ \| 68.40^**^ \| 1375968.43^**^ \| 205.32** \| \| **Genotype (G)** \| 98 \| 15.27^**^ \| 0.49^**^ \| 646.45^**^ \| 11.03^**^ \| 137.12 ^**^ \| 62443.19^**^ \| 95.54^**^ \| 105.58^**^ \| 71963.21^**^ \| 70.80** \| \| **Common wheats** \| 7 \| ^*^ \| ^ns^ \| ^ns^ \| ^ns^ \| ^ns^ \| ^ns^ \| ^**^ \| ^**^ \| ^*^ \| ** \| \| **Synthetic wheats** \| 90 \| ^**^ \| ^ns^ \| ^ns^ \| ^*^ \| ^**^ \| ^**^ \| ^**^ \| ^**^ \| ^**^ \| ** \| \| **Com vs. Syn** \| 1 \| ^**^ \| ^ns^ \| ^*^ \| ^ns^ \| ^*^ \| ^**^ \| ^ns^ \| ^**^ \| ^ns^ \| * \| \| **G * I** \| 98 \| 1.05^*^ \| 0.26 ^ns^ \| 93.45 ^*^ \| 0.12 ^ns^ \| 13.62 ^ns^ \| 3685.16 ^*^ \| 79.55 ^**^ \| 28.07^**^ \| 26774.36 ^ns^ \| 23.73** \| \| **G * Y** \| 98 \| 0.88 ^ns^ \| 0.29 ^ns^ \| 177.45 ^**^ \| 0.02^*^ \| 27.87^**^ \| 3938.33^*^ \| 75.63 ^**^ \| 23.94^*^ \| 50992.67^**^ \| 17.23 ^ns^ \| \| **G * Y * I** \| 98 \| 1.01 ^ns^ \| 0.24 ^ns^ \| 104.56^*^ \| 0.15 ^ns^ \| 17.66 ^ns^ \| 2686.42 ^ns^ \| 84.58^**^ \| 22.66 ^ns^ \| 22354.38 ^ns^ \| 13.76 ^ns^ \| \| **Error** \| 297 \| 0.64 \| 0.23 \| 57.13 \| 1.47 \| 12.04 \| 2140.37 \| 56 \| 19.57 \| 25555.8 \| 14.90 \| \| **R^2^** \|  \| 0.89 \| 0.78 \| 0.89 \| 0.86 \| 0.88 \| 0.90 \| 0.84 \| 0.86 \| 0.88 \| 0.81 \| \| **CV (%)** \|  \| 4.66 \| 5.72 \| 12.48 \| 1.81 \| 5.36 \| 5.73 \| 10.60 \| 12.70 \| 19.09 \| 4.82 \|   **Table S3.** Continued | | | | | | | | | | |  |
| --- | --- | --- | --- | --- | --- | --- | --- | --- | --- | --- | --- | --- | --- | --- | --- | --- | --- | --- | --- | --- | --- | --- | --- | --- | --- | --- | --- | --- | --- | --- | --- | --- | --- | --- | --- | --- | --- | --- | --- | --- | --- | --- | --- | --- | --- | --- | --- | --- | --- | --- | --- | --- | --- | --- | --- | --- | --- | --- | --- | --- | --- | --- | --- | --- | --- | --- | --- | --- | --- | --- | --- | --- | --- | --- | --- | --- | --- | --- | --- | --- | --- | --- | --- | --- | --- | --- | --- | --- | --- | --- | --- | --- | --- | --- | --- | --- | --- | --- | --- | --- | --- | --- | --- | --- | --- | --- | --- | --- | --- | --- | --- | --- | --- | --- | --- | --- | --- | --- | --- | --- | --- | --- | --- | --- | --- | --- | --- | --- | --- | --- | --- | --- | --- | --- | --- | --- | --- | --- | --- | --- | --- | --- | --- | --- | --- | --- | --- | --- | --- | --- | --- | --- | --- | --- | --- | --- | --- | --- | --- | --- | --- | --- | --- | --- | --- | --- | --- | --- | --- | --- | --- | --- | --- | --- | --- | --- | --- | --- | --- | --- | --- | --- | --- | --- | --- | --- | --- | --- | --- | --- | --- | --- | --- | --- | --- | --- | --- | --- | --- | --- | --- | --- | --- | --- | --- | --- | --- | --- | --- | --- | --- | --- | --- |
| **SV** | **df** | **Mean Square** | | | | | | | | |  |
|  |  | **WSRC** | **SCSRC** | **SuSRC** | **LASRC** | **MBSSRC** | **CaCl_2_SRC** | **SDSSRC** | **SDS+MBSSRC** | **EtSRC** | **GPI** |
| **Irrigation (I)** | 1 | 27150.59^*^ | 7112.39^*^ | 8538.50 ^**^ | 8176.50^**^ | 5152.77^**^ | 6350.39^*^ | 10030.12^**^ | 10490.98^*^ | 17339.22^**^ | 4.79 ^ns^ |
| **Year (Y)** | 1 | 47.19 ^ns^ | 498.98 ^ns^ | 1193.30 ^ns^ | 1325.97 ^*^ | 376.44 ^ns^ | 118.35 ^ns^ | 800.28 ^ns^ | 396.58 ^ns^ | 549.52 ^ns^ | 5.66 ^ns^ |
| **Y * I** | 1 | 3013 ^ns^ | 23.59 ^ns^ | 266.76 ^ns^ | 397.23 ^ns^ | 80.62 ^ns^ | 628.37 ^ns^ | 1.54 ^ns^ | 24.48 ^ns^ | 23.69 ^ns^ | 5.56 ^ns^ |
| **Rep (Y * I)** | 8 | 1618.61 ^**^ | 340.20 ^**^ | 207.95 ^*^ | 110.04 ^*^ | 103.20 ^ns^ | 308.37^**^ | 164.91 ^ns^ | 520.41^**^ | 95.61 ^ns^ | 1.24 ^ns^ |
| **Genotype (G)** | 98 | 636.50 ^**^ | 625.01^**^ | 550.66 ^**^ | 374.82^**^ | 637.43^**^ | 537.12^**^ | 467.12^**^ | 1153.28^**^ | 1248.58^**^ | 5.64^**^ |
| **Common wheats** | 7 | ^**^ | ^**^ | ^**^ | ^**^ | ^**^ | ^**^ | ^**^ | ^**^ | ^**^ | ^*^ |
| **Synthetic wheats** | 90 | ^**^ | ^**^ | ^**^ | ^**^ | ^**^ | ^**^ | ^**^ | ^**^ | ^**^ | ^**^ |
| **Com vs. Syn** | 1 | ^ns^ | ^ns^ | ^ns^ | ^ns^ | ^ns^ | ^ns^ | ^ns^ | ^**^ | ^ns^ | ^ns^ |
| **G * I** | 98 | 500.70 ^**^ | 261.44 ^**^ | 312.33 ^**^ | 219.63 ^**^ | 355.75 ^**^ | 151.82 ^**^ | 282.16^**^ | 313.93^**^ | 226.81^**^ | 5.99^**^ |
| **G * Y** | 98 | 8.00 ^ns^ | 64.01 ^ns^ | 134.88 ^*^ | 24.69 ^ns^ | 26.27 ^ns^ | 31.73 ^ns^ | 7.14 ^ns^ | 16.26 ^ns^ | 7.85 ^ns^ | 1.82 ^ns^ |
| **G * Y * I** | 98 | 210.02 ^ns^ | 63.43 ^ns^ | 133.06 ^*^ | 24.67 ^ns^ | 16.97 ^ns^ | 25.31 ^ns^ | 7.98 ^ns^ | 15.89 ^ns^ | 7.15 ^ns^ | 1.78 ^ns^ |
| **Error** | 297 | 220.02 | 80.44 | 77.39 | 43.93 | 80.82 | 70.67 | 72.91 | 62.26 | 54.73 | 1.86 |
| **R^2^** |  | 0.75 | 0.80 | 0.83 | 0.85 | 0.79 | 0.78 | 0.80 | 0.88 | 0.90 | 0.68 |
| **CV (%)** |  | 7.35 | 3.99 | 3.79 | 3.17 | 4.47 | 3.68 | 4.37 | 3.98 | 3.70 | 2.98 |
| ns; *; ** Non-significant, Significant at 0.05 and 0.01 probability level, respectively  RMT rapid mix test (ml/100 gr flour), RWC (%) relative water content, TGW (g) thousand-grain weight, YLD (g/m^2^) grain yield, He hectoliter (kg/he), WSRC Water Solvent retention capacity, SCSRC Sodium carbonate Solvent retention capacity, SuSRC Sucrose Solvent retention capacity, LASRC Lactic acid Solvent retention capacity, MBSSRC Sodium Metabisulfite Solvent retention capacity, CaCl_2_SRC Chloride calcium Solvent retention capacity, SDSSRC Sodium dodecyl sulfate Solvent retention capacity, SDS+MBSSRC Sodium dodecyl sulfate + Sodium Metabisulfite Solvent retention capacity, EtSRC Ethanol Solvent retention capacity, GPI Gluten Performance Index, CV (%) coefficient of variation. | | | | | | | | | | |  |
