# Supplementary material for: Synthetic wheat as a new source of flour quality under drought conditions: Associations with solvent retention capacity
Source: PLoS One. 2025 Feb 6;20(2):e0316945. doi: 10.1371/journal.pone.0316945 (PMC11801611; doi:10.1371/journal.pone.0316945)
Supplement: S4 Table — (DOCX) [file pone.0316945.s004.docx]

**S4 Table. Means of important quality traits in synthetic wheat genotypes under well-irrigated and water stressed conditions during 2018-2019.**

| **Genotype code** | **WSRC** | | **LASRC** | | **MBSSRC** | | **SCSRC** | | **RMT** | | **ZEL** | |
| --- | --- | --- | --- | --- | --- | --- | --- | --- | --- | --- | --- | --- |
|  | **Normal** | **Water stress** | **Normal** | **Water stress** | **Normal** | **Water stress** | **Normal** | **Water stress** | **Normal** | **Water stress** | **Normal** | **Water stress** |
| 1 | 197.15 | 214.62 | 231.15 | 236.25 | 206.19 | 218.03 | 195.58 | 202.50 | 74.25 | 70.00 | 862.75 | 902.00 |
| 2 | 192.49 | 200.75 | 218.65 | 219.41 | 193.29 | 206.31 | 191.97 | 200.71 | 69.75 | 69.00 | 899.75 | 929.25 |
| 4 | 198.31 | 207.76 | 222.73 | 225.63 | 209.87 | 208.48 | 201.80 | 197.81 | 62.75 | 57.25 | 830.00 | 848.00 |
| 5 | 185.07 | 206.84 | 214.05 | 215.11 | 194.73 | 213.51 | 193.02 | 189.18 | 65.75 | 60.25 | 865.00 | 881.75 |
| 7 | 200.35 | 220.06 | 224.37 | 248.55 | 206.44 | 226.87 | 203.22 | 227.93 | 62.00 | 67.75 | 911.25 | 881.50 |
| 8 | 191.05 | 212.72 | 222.47 | 233.53 | 205.55 | 212.40 | 198.19 | 215.37 | 58.50 | 65.75 | 883.25 | 923.25 |
| 14 | 192.79 | 198.71 | 219.83 | 231.60 | 203.77 | 206.59 | 195.52 | 200.69 | 67.00 | 70.50 | 849.25 | 884.00 |
| 15 | 194.43 | 189.22 | 202.29 | 222.67 | 199.39 | 207.98 | 187.86 | 192.55 | 52.50 | 52.50 | 860.50 | 860.50 |
| 16 | 192.21 | 192.29 | 206.11 | 225.74 | 195.56 | 204.27 | 181.81 | 196.89 | 66.00 | 66.00 | 757.50 | 757.50 |
| 17 | 190.97 | 200.54 | 215.58 | 224.31 | 202.03 | 212.02 | 191.82 | 198.32 | 71.75 | 68.25 | 854.00 | 888.50 |
| 21 | 188.80 | 197.63 | 214.64 | 230.33 | 199.70 | 212.46 | 186.79 | 206.78 | 50.00 | 50.00 | 893.50 | 893.50 |
| 23 | 180.36 | 183.99 | 200.10 | 200.92 | 187.78 | 190.16 | 178.95 | 189.42 | 62.00 | 62.00 | 542.50 | 542.50 |
| 25 | 179.14 | 193.31 | 194.47 | 204.29 | 187.55 | 193.57 | 178.19 | 189.50 | 49.50 | 49.50 | 713.00 | 713.00 |
| 27 | 185.03 | 193.05 | 206.33 | 209.10 | 194.71 | 201.16 | 188.06 | 187.51 | 55.75 | 59.00 | 752.25 | 794.25 |
| 29 | 185.99 | 196.36 | 215.92 | 227.59 | 193.20 | 199.83 | 186.70 | 191.90 | 55.50 | 55.50 | 904.00 | 904.00 |
| 30 | 180.07 | 183.37 | 195.84 | 209.47 | 189.76 | 189.74 | 179.58 | 185.59 | 54.00 | 54.00 | 693.00 | 693.00 |
| 33 | 193.38 | 191.07 | 210.05 | 190.50 | 197.09 | 182.59 | 186.63 | 190.02 | 54.50 | 54.50 | 697.50 | 697.50 |
| 34 | 192.56 | 219.17 | 211.13 | 232.37 | 198.66 | 209.18 | 186.11 | 213.50 | 50.50 | 50.50 | 672.00 | 672.00 |
| 35 | 196.34 | 204.61 | 209.56 | 219.30 | 199.56 | 211.55 | 188.72 | 200.31 | 50.00 | 50.00 | 700.50 | 700.50 |
| 43 | 198.73 | 206.16 | 208.84 | 235.66 | 193.28 | 209.19 | 188.54 | 201.72 | 60.50 | 60.50 | 770.50 | 770.50 |
| 48 | 187.64 | 191.17 | 203.70 | 213.78 | 195.85 | 201.26 | 187.44 | 188.25 | 69.50 | 69.50 | 952.00 | 952.00 |
| 50 | 189.89 | 196.86 | 218.76 | 232.58 | 202.28 | 205.25 | 192.37 | 196.55 | 58.00 | 58.00 | 819.00 | 819.00 |
| 53 | 201.03 | 214.59 | 211.07 | 232.73 | 195.54 | 211.23 | 192.88 | 209.91 | 56.50 | 56.50 | 712.50 | 712.50 |
| 54 | 178.33 | 190.48 | 207.86 | 199.97 | 198.51 | 191.17 | 189.78 | 186.59 | 53.50 | 52.50 | 739.50 | 800.50 |
| 55 | 196.05 | 103.88 | 218.70 | 231.02 | 198.31 | 210.42 | 196.15 | 197.35 | 53.50 | 53.50 | 802.50 | 802.50 |
| 58 | 189.48 | 193.42 | 220.71 | 210.40 | 202.22 | 204.94 | 198.90 | 193.76 | 55.75 | 61.25 | 632.75 | 723.00 |
| 62 | 192.40 | 232.29 | 222.04 | 238.26 | 192.15 | 233.19 | 187.03 | 227.38 | 50.50 | 50.50 | 665.50 | 665.50 |
| 63 | 187.92 | 192.98 | 201.11 | 218.42 | 187.81 | 209.10 | 180.78 | 187.46 | 66.50 | 66.50 | 904.00 | 904.00 |
| 65 | 185.39 | 202.85 | 223.63 | 239.56 | 201.34 | 213.92 | 190.51 | 206.15 | 58.50 | 53.25 | 648.50 | 774.75 |
| 66 | 200.49 | 278.24 | 226.10 | 262.05 | 206.23 | 278.64 | 203.22 | 240.57 | 57.50 | 57.50 | 902.00 | 902.00 |
| 67 | 181.23 | 233.72 | 199.44 | 240.18 | 186.21 | 222.89 | 184.40 | 219.87 | 62.50 | 62.50 | 773.50 | 773.50 |
| 68 | 189.99 | 205.62 | 212.74 | 222.30 | 193.21 | 212.57 | 189.22 | 194.48 | 60.50 | 60.50 | 912.50 | 912.50 |
| 71 | 187.95 | 194.55 | 204.53 | 207.86 | 190.09 | 207.83 | 184.29 | 190.72 | 58.50 | 58.50 | 660.00 | 660.00 |
| 72 | 171.95 | 196.96 | 192.09 | 219.78 | 182.98 | 213.56 | 188.23 | 190.92 | 55.00 | 55.00 | 766.00 | 766.00 |
| 73 | 190.81 | 274.07 | 213.94 | 260.62 | 197.22 | 264.91 | 192.06 | 277.39 | 46.50 | 46.50 | 889.00 | 889.00 |
| 74 | 181.69 | 193.64 | 192.88 | 210.02 | 185.15 | 201.92 | 182.90 | 186.76 | 48.50 | 48.50 | 656.50 | 656.50 |
| 77 | 180.31 | 198.28 | 185.34 | 207.49 | 180.96 | 210.17 | 176.65 | 192.76 | 59.50 | 59.50 | 641.00 | 641.00 |
| 78 | 182.66 | 185.69 | 196.95 | 191.66 | 189.28 | 199.12 | 183.83 | 181.06 | 82.00 | 82.00 | 758.00 | 758.00 |
| 79 | 190.05 | 191.77 | 209.50 | 209.05 | 198.36 | 201.60 | 188.78 | 187.03 | 63.00 | 63.00 | 727.50 | 727.50 |
| 80 | 182.58 | 218.71 | 212.13 | 223.03 | 198.32 | 215.17 | 187.55 | 204.08 | 66.75 | 63.75 | 730.75 | 795.75 |
| 82 | 199.31 | 214.50 | 224.97 | 229.29 | 210.50 | 206.37 | 205.42 | 199.787 | 65.50 | 57.00 | 778.75 | 821.00 |
| 85 | 190.20 | 199.26 | 220.91 | 230.37 | 205.41 | 202.04 | 201.70 | 197.181 | 62.00 | 59.75 | 740.50 | 788.25 |
| 86 | 176.83 | 193.93 | 188.28 | 213.15 | 181.33 | 207.26 | 169.93 | 185.191 | 74.00 | 74.00 | 943.00 | 943.00 |
| 88 | 195.57 | 210.62 | 208.99 | 216.36 | 197.91 | 215.85 | 175.45 | 196.665 | 59.00 | 59.00 | 697.00 | 697.00 |
| 89 | 189.17 | 210.04 | 205.11 | 234.63 | 195.23 | 215.34 | 185.26 | 199.053 | 50.50 | 50.50 | 718.50 | 718.50 |
| 94 | 193.89 | 205.56 | 222.14 | 240.75 | 200.26 | 247.40 | 194.46 | 215.818 | 53.50 | 53.50 | 812.00 | 812.00 |
| 98 | 191.18 | 183.70 | 218.81 | 201.92 | 203.55 | 197.56 | 200.25 | 176.12 | 40.50 | 40.50 | 832.00 | 832.00 |
| 99 | 188.84 | 188.81 | 204.89 | 207.24 | 202.53 | 215.62 | 186.74 | 187.76 | 45.00 | 45.00 | 661.00 | 661.00 |
| 102 | 190.53 | 226.49 | 210.27 | 234.23 | 198.24 | 218.03 | 194.74 | 210.11 | 58.50 | 60.50 | 748.25 | 769.75 |
| 105 | 191.09 | 229.21 | 215.05 | 246.08 | 210.48 | 248.90 | 191.91 | 222.59 | 53.50 | 53.50 | 698.00 | 698.00 |
| 107 | 198.73 | 439.49 | 219.47 | 268.91 | 219.15 | 289.16 | 194.54 | 270.32 | 51.00 | 51.00 | 740.00 | 740.00 |
| 109 | 226.25 | 218.38 | 233.36 | 255.21 | 230.30 | 256.69 | 213.75 | 223.57 | 52.50 | 52.50 | 744.00 | 744.00 |
| 110 | 202.13 | 226.06 | 224.46 | 257.66 | 220.19 | 235.25 | 220.95 | 242.04 | 40.00 | 40.00 | 897.00 | 897.00 |
| 111 | 175.12 | 199.19 | 179.43 | 236.77 | 201.99 | 224.71 | 178.74 | 195.90 | 55.50 | 55.50 | 727.00 | 727.00 |
| 115 | 176.46 | 191.65 | 195.56 | 228.86 | 184.44 | 217.65 | 175.66 | 193.22 | 50.00 | 50.00 | 744.00 | 744.00 |
| 116 | 191.78 | 197.75 | 202.55 | 236.05 | 201.26 | 223.21 | 187.02 | 198.45 | 58.00 | 58.00 | 710.00 | 710.00 |
| 118 | 189.94 | 217.57 | 208.31 | 234.84 | 198.52 | 222.57 | 187.31 | 211.27 | 49.50 | 49.50 | 731.50 | 731.50 |
| 120 | 187.54 | 201.93 | 200.66 | 229.11 | 193.41 | 219.30 | 188.05 | 196.12 | 51.50 | 51.50 | 632.50 | 632.50 |
| 122 | 198.83 | 194.28 | 222.29 | 226.37 | 207.94 | 213.58 | 196.06 | 196.44 | 64.00 | 64.00 | 812.00 | 812.00 |
| 123 | 199.45 | 198.25 | 207.48 | 216.13 | 196.64 | 209.60 | 189.71 | 200.01 | 42.50 | 42.50 | 658.50 | 658.50 |
| 124 | 198.78 | 191.97 | 227.38 | 234.44 | 226.61 | 202.87 | 200.69 | 199.28 | 46.50 | 46.50 | 881.50 | 881.50 |
| 132 | 195.04 | 211.12 | 210.32 | 215.29 | 218.42 | 205.86 | 196.40 | 199.37 | 46.00 | 46.00 | 662.00 | 662.00 |
| 133 | 190.26 | 196.02 | 206.41 | 207.82 | 202.42 | 205.75 | 191.68 | 196.61 | 56.50 | 56.50 | 664.00 | 664.00 |
| 135 | 192.84 | 208.96 | 226.30 | 235.37 | 200.64 | 214.71 | 196.90 | 201.89 | 55.75 | 59.50 | 742.00 | 817.50 |
| 137 | 196.30 | 229.18 | 227.83 | 231.43 | 210.03 | 227.77 | 192.90 | 210.83 | 44.50 | 44.50 | 733.00 | 733.00 |
| 139 | 196.63 | 185.60 | 215.34 | 206.35 | 201.51 | 200.41 | 189.69 | 184.84 | 48.00 | 48.00 | 871.00 | 871.00 |
| 141 | 188.25 | 210.14 | 200.49 | 241.19 | 197.09 | 217.09 | 185.96 | 232.20 | 74.50 | 74.50 | 776.50 | 776.50 |
| 142 | 191.08 | 193.67 | 206.60 | 210.39 | 194.95 | 198.54 | 187.62 | 189.01 | 49.00 | 49.00 | 652.00 | 652.00 |
| 143 | 188.00 | 187.43 | 195.88 | 201.72 | 192.87 | 194.16 | 181.36 | 185.06 | 47.00 | 47.00 | 587.00 | 587.00 |
| 144 | 193.92 | 192.09 | 213.36 | 227.13 | 197.07 | 200.50 | 191.10 | 187.81 | 52.00 | 52.00 | 760.50 | 760.50 |
| 152 | 191.41 | 262.87 | 212.10 | 249.02 | 200.46 | 227.51 | 191.99 | 227.10 | 57.00 | 57.00 | 744.50 | 744.50 |
| 154 | 193.03 | 239.52 | 215.67 | 254.73 | 206.50 | 230.98 | 194.60 | 219.88 | 57.75 | 53.25 | 699.75 | 811.25 |
| 157 | 202.60 | 200.07 | 219.54 | 229.44 | 204.93 | 210.50 | 197.60 | 195.37 | 42.00 | 42.00 | 882.50 | 882.50 |
| 158 | 193.22 | 210.70 | 199.26 | 226.01 | 192.00 | 208.78 | 189.55 | 199.12 | 47.50 | 47.50 | 651.50 | 651.50 |
| 159 | 194.75 | 234.43 | 223.30 | 230.31 | 205.20 | 216.00 | 201.61 | 206.28 | 46.75 | 52.25 | 732.50 | 729.75 |
| 161 | 188.51 | 188.63 | 222.24 | 225.52 | 192.16 | 205.58 | 188.61 | 190.21 | 56.00 | 56.00 | 793.50 | 793.50 |
| 166 | 202.45 | 201.31 | 210.88 | 220.80 | 194.49 | 200.44 | 192.31 | 193.74 | 45.00 | 45.00 | 626.50 | 626.50 |
| 167 | 188.19 | 192.73 | 205.11 | 215.87 | 192.24 | 202.66 | 188.69 | 184.63 | 40.50 | 40.50 | 664.00 | 664.00 |
| 168 | 191.16 | 199.83 | 228.52 | 237.85 | 204.26 | 205.74 | 193.02 | 194.65 | 45.50 | 45.50 | 835.00 | 835.00 |
| 169 | 190.48 | 221.28 | 223.42 | 253.44 | 200.29 | 227.80 | 191.03 | 210.44 | 44.50 | 44.50 | 878.50 | 878.50 |
| 170 | 187.11 | 211.86 | 224.23 | 243.24 | 199.30 | 229.99 | 191.90 | 203.09 | 59.00 | 59.00 | 761.00 | 761.00 |
| 173 | 188.62 | 207.72 | 210.49 | 221.35 | 195.48 | 206.60 | 196.20 | 197.15 | 52.50 | 48.50 | 703.75 | 787.50 |
| 174 | 196.84 | 193.49 | 230.08 | 229.04 | 208.36 | 209.25 | 198.84 | 193.24 | 49.00 | 49.00 | 766.50 | 766.50 |
| 178 | 202.74 | 234.46 | 237.40 | 257.62 | 220.34 | 218.24 | 200.70 | 238.10 | 52.00 | 52.00 | 807.00 | 807.00 |
| 181 | 194.39 | 213.81 | 216.56 | 239.24 | 203.27 | 212.60 | 201.75 | 216.60 | 49.00 | 49.00 | 798.50 | 798.50 |
| 183 | 196.87 | 198.75 | 221.10 | 225.18 | 214.91 | 198.82 | 193.87 | 197.90 | 49.00 | 49.00 | 811.00 | 811.00 |
| 184 | 196.64 | 190.50 | 219.88 | 217.72 | 207.64 | 197.98 | 194.40 | 211.30 | 56.50 | 56.50 | 716.00 | 716.00 |
| 185 | 198.14 | 209.68 | 221.48 | 234.83 | 214.35 | 216.70 | 198.91 | 201.63 | 58.00 | 58.00 | 786.00 | 786.00 |
| 188 | 186.39 | 187.01 | 206.82 | 203.18 | 194.75 | 184.83 | 186.62 | 175.52 | 55.00 | 55.00 | 717.00 | 717.00 |
| 192 | 187.36 | 186.02 | 210.62 | 216.42 | 190.49 | 195.76 | 187.89 | 186.15 | 52.00 | 52.00 | 814.50 | 814.50 |
| 196 | 195.94 | 206.87 | 218.80 | 234.84 | 206.73 | 217.48 | 195.74 | 200.21 | 57.75 | 62.75 | 717.75 | 768.50 |
| 197 | 195.42 | 198.61 | 215.65 | 231.81 | 195.60 | 214.57 | 195.55 | 201.60 | 51.00 | 51.00 | 717.50 | 717.50 |
| 198 | 188.17 | 194.37 | 212.58 | 220.30 | 205.14 | 212.09 | 191.43 | 192.24 | 57.25 | 57.75 | 702.50 | 768.50 |
| 199 | 194.58 | 212.81 | 222.76 | 240.31 | 203.10 | 220.37 | 188.67 | 204.89 | 55.00 | 63.25 | 727.25 | 845.75 |
| 200 | 178.71 | 225.88 | 216.77 | 233.20 | 205.46 | 212.28 | 194.71 | 204.95 | 59.50 | 65.50 | 812.25 | 898.75 |
| 205 | 188.61 | 259.38 | 216.22 | 245.28 | 205.98 | 232.66 | 202.42 | 256.87 | 60.75 | 66.50 | 751.75 | 799.75 |
| 206 | 187.46 | 209.30 | 220.87 | 223.29 | 206.47 | 224.79 | 195.45 | 200.51 | 47.50 | 55.25 | 745.50 | 851.00 |
| 207 | 193.92 | 234.21 | 226.01 | 261.78 | 202.95 | 227.33 | 203.18 | 231.47 | 67.00 | 57.00 | 873.00 | 943.00 |
| 208 | 191.73 | 219.75 | 226.44 | 236.35 | 206.32 | 247.11 | 197.11 | 220.36 | 66.75 | 60.50 | 891.00 | 948.50 |
