# Supplementary material for: Synthetic wheat as a new source of flour quality under drought conditions: Associations with solvent retention capacity
Source: PLoS One. 2025 Feb 6;20(2):e0316945. doi: 10.1371/journal.pone.0316945 (PMC11801611; doi:10.1371/journal.pone.0316945)
Supplement: S1 Fig — Correlation coefficients among different quality traits and tolerance index (CSI) of SHW population evaluated normal (a) and water stress (b) conditions. WSRC Water Solvent retention capacity, SCSRC Sodium carbonate Solvent retention capacity, LASRC Lactic acid Solvent retention capacity, SuSRC Sucrose Solvent retention capacity, MBSSRC Sodium Metabisulfite Solvent retention capacity, CaCl2SRC Chloride calcium Solvent retention capacity, SDSSRC Sodium dodecyl sulfate Solvent retention capacity, SDS+MBSSRC Sodium Metabisulfite Sodium dodecyl sulfate + Solvent retention capacity, EtSRC Ethanol Solvent retention capacity, PRO Protein, MOI Moisture, ZEL Zeleny, WABS Water Absorbance, HAR Hardness, RMT rapid mix test (ml/100 gr flour), TGW (g) thousand-grain weight, RWC Relative water content, He hectoliter (kg/he), GY (g/m2) grain yield, CSI Combination of significant index. (DOCX) [file pone.0316945.s005.docx]

| 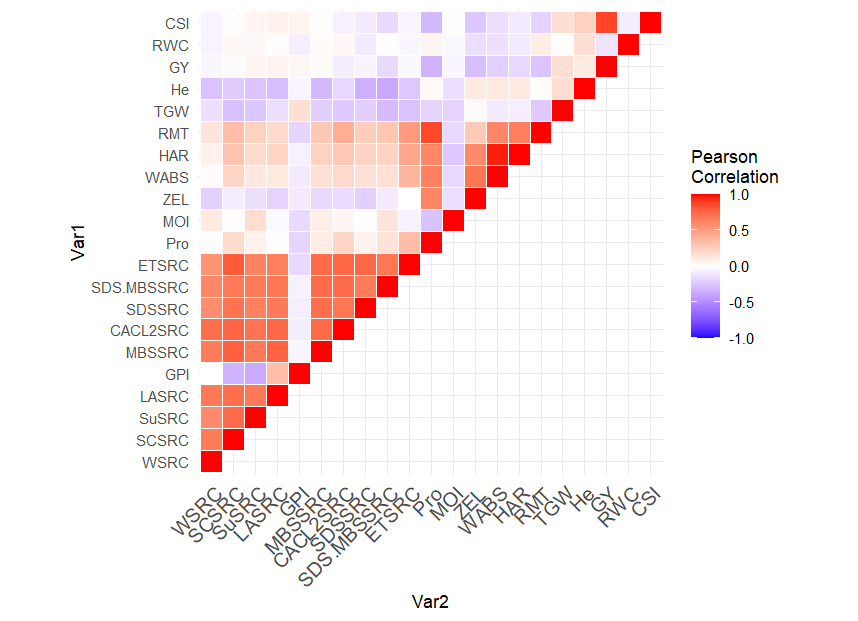 |
| --- |
| 1. Normal |
| 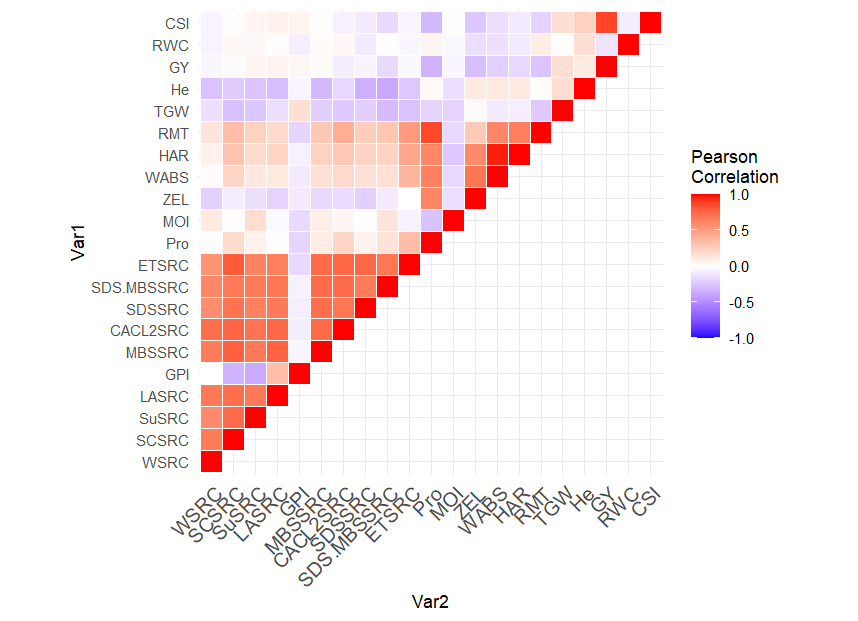 |
| 1. Water stress |
| **S1 Fig.** Correlation coefficients among different quality traits and tolerance index (CSI) of SHW population evaluated normal (a) and water stress (b) conditions. WSRC Water Solvent retention capacity, SCSRC Sodium carbonate Solvent retention capacity, LASRC Lactic acid Solvent retention capacity, SuSRC Sucrose Solvent retention capacity, MBSSRC Sodium Metabisulfite Solvent retention capacity, CaCl2SRC Chloride calcium Solvent retention capacity, SDSSRC Sodium dodecyl sulfate Solvent retention capacity, SDS+MBSSRC Sodium Metabisulfite Sodium dodecyl sulfate + Solvent retention capacity, EtSRC Ethanol Solvent retention capacity, PRO Protein, MOI Moisture, ZEL Zeleny, WABS Water Absorbance, HAR Hardness, RMT rapid mix test (ml/100 gr flour), TGW (g) thousand-grain weight, RWC Relative water content, He hectoliter (kg/he), GY (g/m^2^) grain yield, CSI Combination of significant index. |
